# Localization of beta power decrease as measure for lateralization in pre-surgical language mapping with magnetoencephalography, compared with functional magnetic resonance imaging and validated by Wada test

**DOI:** 10.3389/fnhum.2022.996989

**Published:** 2022-10-26

**Authors:** Kirsten Herfurth, Yuval Harpaz, Julie Roesch, Nadine Mueller, Katrin Walther, Martin Kaltenhaeuser, Elisabeth Pauli, Abraham Goldstein, Hajo Hamer, Michael Buchfelder, Arnd Doerfler, Julian Prell, Stefan Rampp

**Affiliations:** ^1^Department of Neurosurgery, University Hospital Erlangen, Erlangen, Germany; ^2^Department of Neurosurgery, University Hospital Halle, Halle (Saale), Germany; ^3^The Gonda Multidisciplinary Brain Research Center, Bar-Ilan University, Ramat Gan, Israel; ^4^Department of Neuroradiology, University Hospital Erlangen, Erlangen, Germany; ^5^Epilepsy Center, Department of Neurology, University Hospital Erlangen, Erlangen, Germany

**Keywords:** language lateralization, magnetoencephalography (MEG), functional magnetic resonance imaging (fMRI), epilepsy, epilepsy surgery, intracarotid sodium amobarbital procedure (IAT), Wada test, beta power decrease

## Abstract

**Objective:** Atypical patterns of language lateralization due to early reorganizational processes constitute a challenge in the pre-surgical evaluation of patients with pharmaco-resistant epilepsy. There is no consensus on an optimal analysis method used for the identification of language dominance in MEG. This study examines the concordance between MEG source localization of beta power desynchronization and fMRI with regard to lateralization and localization of expressive and receptive language areas using a visual verb generation task.

**Methods:** Twenty-five patients with pharmaco-resistant epilepsy, including six patients with atypical language lateralization, and ten right-handed controls obtained MEG and fMRI language assessment. Fourteen patients additionally underwent the Wada test. We analyzed MEG beta power desynchronization in sensor (controls) and source space (patients and controls). Beta power decrease between 13 and 35 Hz was localized applying Dynamic Imaging of Coherent Sources Beamformer technique. Statistical inferences were grounded on cluster-based permutation testing for single subjects.

**Results:** Event-related desynchronization of beta power in MEG was seen within the language-dominant frontal and temporal lobe and within the premotor cortex. Our analysis pipeline consistently yielded left language dominance with high laterality indices in controls. Language lateralization in MEG and Wada test agreed in all 14 patients for inferior frontal, temporal and parietal language areas (Cohen’s Kappa = 1, *p* < 0.001). fMRI agreed with Wada test in 12 out of 14 cases (85.7%) for Broca’s area (Cohen’s Kappa = 0.71, *p* = 0.024), while the agreement for temporal and temporo-parietal language areas were non-significant. Concordance between MEG and fMRI laterality indices was highest within the inferior frontal gyrus, with an agreement in 19/24 cases (79.2%), and non-significant for Wernicke’s area. Spatial agreement between fMRI and MEG varied considerably between subjects and brain regions with the lowest Euclidean distances within the inferior frontal region of interest.

**Conclusion:** Localizing the desynchronization of MEG beta power using a verb generation task is a promising tool for the identification of language dominance in the pre-surgical evaluation of epilepsy patients. The overall agreement between MEG and fMRI was lower than expected and might be attributed to differences within the baseline condition. A larger sample size and an adjustment of the experimental designs are needed to draw further conclusions.

## Introduction

Patients with intractable temporal or frontal lobe epilepsy undergoing surgery are at risk of post-operative language function decline. Variable patterns of language lateralization and localization due to early reorganizational processes constitute a considerable challenge in the pre-surgical evaluation of patients with pharmaco-resistant epilepsy (Springer et al., 1999; Berl et al., 2014). Thus, the presurgical evaluation of patients includes the assessment of language lateralization and localization in order to spare the eloquent cortex. The use of the invasive Wada test, also known under the term “intra-carotid sodium amobarbital procedure” (IAT), for language lateralization has considerably decreased since the emergence of non-invasive fMRI (Kundu et al., 2019). Nevertheless, the Wada test has remained an essential tool in the evaluation of selected patients with epilepsy (Kundu et al., 2019) and is still considered the gold standard of language lateralization (Bauer et al., 2014).

fMRI, which enables the investigation of the functional organization of language areas in the brain by taking advantage of the BOLD (Blood Oxygenation Level Dependent) effect, benefits from its high spatial resolution and the high availability of MRI scanners in clinical practice. While agreement rates with the Wada test are high in patients with strong left-lateralized language dominance, discordant fMRI lateralization is seen in patients with atypical Wada test outcomes in up to 50% of the cases (Janecek et al., 2013). An extension of the meta-analysis by Bauer et al. (2014) including 31 studies revealed sensitivity scores ranging between 64.3% and 100% and specificity values ranging from 28.6% to 100%, with an overall sensitivity of 88.8%, and an overall specificity of 74.1% (Massot-Tarrús et al., 2017). While robust evidence is reported for patients with circumscribed temporal lobe epilepsy (TLE), less reliable results are found in patients with extra-temporal foci, temporal neo-cortical epilepsy, more extended lesions, or in patients with impaired cognitive performance (Szaflarski et al., 2017). Open research questions also regard the influence of the underlying pathology on the BOLD effect. For instance, Nadkarni et al. (2015) found differences in activation patterns in patients with vascular lesions indicating abnormal flow dynamics which complicate the interpretation of fMRI even in tissue structures adjacent to the lesion that might also be susceptible to hypoperfusion.

MEG measures changes in the magnetic fields originating from neural electrical currents. In comparison to fMRI, MEG allows the direct recording of neuronal activation, and is thus independent of cerebral blood flow abnormalities and is suited for patients with tumors and other malformations (Kamada et al., 2007). The high temporal resolution of less than 1 ms permits the investigation of the temporal dynamics of the neural network associated with cognitive processing. MEG has the advantage of measuring patients in a silent setting in either seated or supine position, with a seated position being helpful for maintaining attention and performance in long and monotonous experiments. MEG has further been proven as suitable for children (Fisher et al., 2008) as well as for patients with anxiety disorders (Rich et al., 2010).

Studies investigating the concordance between MEG and Wada test for language lateralization reveal comparable patterns to fMRI findings. Overall concordance rates in 37 analyzed studies published until 2009 range between 71% and 100% (Pirmoradi et al., 2010). Another study reports sensitivity and specificity of 100% and 93% for receptive language areas, as well as a sensitivity and specificity of 83% and 93% for expressive language areas, respectively (Findlay et al., 2012). Tanaka et al. (2013) showed an agreement in 91.4% of cases. Atypical language representation in Wada test, or interference of interictal activity was made responsible for lower overall concordance rates (Papanicolaou et al., 2004; Doss et al., 2009). In a study by Doss et al. (2009), an agreement of 60% was achieved when investigating solely patients with atypical Wada test outcomes.

MEG exhibits a good capability to lateralize receptive language areas (Kamada et al., 1998, 2007; Papanicolaou et al., 1999, 2004). In contrast, language areas in the frontal lobe were found to be less activated, independent of the usage of receptive or expressive language tasks (Pirmoradi et al., 2010).

Apart from the sensitivity of MEG to artifacts, discordant rates in previous studies might be influenced by a wide spectrum of degrees of freedom inherent to MEG analysis, including the chosen source estimation procedure, the analyzed time bins, and the frequency range of the electromagnetic power spectrum. While most studies analyzed broadband electromagnetic activity encompassing frequencies ranging from 1 to 50 Hz or higher (e.g., Tanaka et al., 2013; Kemp et al., 2018), frequencies within the beta band (13–30 Hz) were revealed to be of particular importance during language processing (Weiss and Mueller, 2012). In particular, a decrease in beta band activity, also referred to as event-related desynchronization, demonstrated high concordance with left language lateralization (Pang et al., 2011; Youssofzadeh et al., 2020) as well as fMRI (Foley et al., 2019) and Wada test results (Hirata et al., 2004, 2010; Fisher et al., 2008; Findlay et al., 2012). Beta desynchronization during verb generation tasks was a very robust marker for assessing language lateralization, achieving a sensitivity of 100% in atypical Wada test patients (Fisher et al., 2008; Findlay et al., 2012).

This study intends to validate a MEG protocol for language lateralization and localization and compare it to fMRI and Wada test lateralization results which are used for pre-surgical language mapping at the Epilepsy Center Erlangen/Germany. We analyzed beta power changes during a visual verb generation task to provide localization and lateralization of language-related cortical areas.

Our protocol was first tested in a group of 10 healthy subjects, and subsequently analyzed in 25 patients with intractable temporal or extra-temporal epilepsy undergoing pre-surgical evaluation at our center. MEG lateralization patterns were validated against fMRI and Wada test results.

## Materials and Methods

### Patient group

We retrospectively analyzed data from 25 adult patients with medically intractable epilepsy who underwent MEG and fMRI language assessment during pre-surgical evaluation at the Epilepsy Center Erlangen between the years 2012 and 2013. All patients gave their written informed consent to use their anonymized data for scientific purposes and publication. A total of 15 of these patients underwent Wada testing. The decision for Wada test was based on the neuropsychological indication of atypical memory or language organization and its risk of postsurgical decline. Wada testing and fMRI were aborted due to anxiety in one patient, while MEG language assessment was tolerated by her. Due to the non-availability of fMRI and Wada test results, she was excluded from the study. Five patients underwent epilepsy surgery, in three cases within the dominant hemisphere. We have neuropsychological follow-up data in four of these patients.

The demographic and clinical characteristics of the investigated 24 patients are summarized in Table 1. The mean age was 41.6 years; the mean age at seizure onset was 22.5 years. Four patients were left-handed and three patients were ambidexters. A total of 10 patients were diagnosed with hippocampus sclerosis, three patients with focal cortical dysplasia, one with cortical malformation, three patients were diagnosed with a tumor, and five patients were non-lesional in MRI. In nine patients, the epileptogenic focus was situated within the dominant hemisphere.

**Table 1:** Demographics and clinical characteristics of the patient group.

**ID**	**Age (yrs)**	**Sex**	**Handed-ness**	**Seizure onset (yrs)**	**Language dominance (Wada test)**	**Lesion type and location (MRI)**	**Affected hemisphere**	**Surgery**	**BNT pre OP (% error rate)**	**BNT post OP (% error rate)**
**1**	37	f	ambidexter	15	R	HS	L	-		
**2**	48	m	R	44	L	HS	L	X	18.3	31.7
**3**	57	f	L	25	L	HS	L	X	31.7	41.7
**4**	53	m	R	40	L	HS	L	X	5	8
**5**	28	m	R	2	-	FCD in gyrus temporalis inf., parahippocampal	L	-		
**6**	21	m	R	4	R/L*	Non-lesional	R	-		
**7**	48	f	L	14	-	Cortical malformation of fronto-temporo-parietal cortex, PMG in frontal, insula, temporal lobe, volume reduced hippocampus	L	-	15		
**8**	27	m	R	15	Bil/R^†^	HS	L	X	6.7	10
**9**	34	m	R	12	-	Cavernoma in TL	R	-		
**10**	49	m	R	37	R	Non-lesional	R	-		
**11**	43	m	R	4	-	FCD in gyrus frontalis inferior	R	-	18.3		
**12**	43	f	ambidexter	30	L	HS	L	-	15		
**13**	30	m	R	7	L	Non-lesional	L	-	14		
**14**	51	f	L	24	R	FCD fronto-parietal	L	X	10	Not available
**15**	43	m	R	42	-	Fibrillar astrozytoma grade II, temporo-parietal	L	-		
**16**	22	f	R	21	-	Tumor temporal basal	L	-		
**17**	42	f	R	41	L	Tumor	L	-	30		
**18**	40	m	ambidexter	37	-	HS	R	-		
**19**	59	f	R	53	-	Inflammatory process in temporal lobe	L	-		
**20**	55	f	R	23	L	Non-lesional	Bilateral	-	10		
**21**	41	f	R	1	R	HS	L	-		
**22**	28	f	R	1	-	HS	L	-		
**23**	52	m	R	47	L	Non-lesional	R	-		
**24**	48	f	L	1	-	Extensive post-ischamic defect, secondary Hippocampus Sclerosis	L	-		

*Right hemispheric language dominance with partial left hemispheric language comprehension; ^†^bilateral language comprehension with marked emphasis of the right hemisphere; BNT, Boston Naming Test.

### Control group

We additionally evaluated the MEG and fMRI protocols in 10 healthy volunteers aged 22–41 years (*M* = 31.7, SD = 8.0). Selection criteria were right-handedness in the Edinburgh Handedness Inventory (EHI) test (Oldfield, 1971), normal MRI, and the absence of neurological or psychiatric disorders in the volunteer’s biography. The mean EHI-score was 0.86 (SD = 0.11). One participant was excluded retrospectively due to suspected epilepsy, newly diagnosed after data acquisition. All participants provided written informed consent for the data to be used in the study. This study was carried out in accordance with The Code of Ethics of the World Medical Association (Declaration of Helsinki) for experiments involving humans. The study protocol was approved by the institutional ethics committee.

### Data acquisition and language tasks

Functional imaging was performed using a covert visual verb generation task for both fMRI and MEG. Language lateralization using fMRI is a constituent part of the routine language assessment at the Erlangen Epilepsy Center since 2012. The fMRI language protocol was adopted for MEG, using a different word list.

The decision for implementing a verb generation task in routine fMRI was based on reports of successful application in other centers (e.g., Holland et al., 2001; Rutten et al., 2002). Verb generation also led to successful lateralization of both expressive and receptive language areas in epilepsy patients using MEG (see review of Pirmoradi et al., 2010). Bowyer et al. (2005) reported high robustness of verb generation tasks in detecting atypical language dominance.

#### MEG

MEG language assessment was performed with a 248-channel whole-head MEG-system equipped with magnetometers (Magnes 3600 WHS, 4D Neuroimaging, San Diego, CA). Subjects were measured in seated position, with the participants’ head stabilized using cushions. Position of the subject’s head was recorded using five head coils placed on nasion, left and right auricular points, vertex, and inion.

During the experiment, four randomized blocks with 44 one- or two-syllable words were projected on a mirror system with a distance of 60 cm relative to the subject’s eyes. Black words with gray backgrounds were presented for 1 s, following a fixation cross. As epilepsy patients strongly varied in their verbal performance and speed, we did not use fixed inter-stimulus intervals. Instead, participants were instructed to press a button in order to proceed to the next stimulus after covertly producing a verb. Usage of left and right hand was counterbalanced within subjects. Participants were asked to blink in provided intervals only. In comparison to fMRI, we did not implement a control task in MEG, as pre-stimulus baseline interval can serve as a contrast condition (see Youssofzadeh and Babajani-Feremi, 2019 for discussion). MEG signals were digitally recorded with a 0.1–400 Hz online band pass filter and a sampling rate of 1,017.25 Hz. Recording time varied between 15 and 45 min. In order to confirm adequate performance, all subjects received an overt training session before MEG data acquisition using a separate list of nouns.

#### fMRI

Imaging was performed on a 3T MAGNETOM TIM Trio (Siemens, Erlangen, Germany) using a standard 12-channel head coil. Foam paddings were used to stabilize the subject’s head. Anatomical images were acquired using a T1-weighted 3D MPRAGE sequence (1 mm isotropic resolution, TR/TE/FA = 1,900/2.25/9, Field of view 25.6 cm × 25.6 cm × 25.6 cm). Functional data depicting Blood Oxygen Level Dependent (BOLD) contrast were measured using a 2D echo planar sequence with the following parameters: 1.5 mm × 1.5 mm in plane resolution, 3 mm slice thickness, 0, 75 mm interslice gap, TR/TE/FA = 3,000/30/90, ascending and interleaved acquisition, FOV = 192 mm, 128 × 128 matrix. To allow signal stabilization, the initial volumes of the fMRI acquisition were automatically removed by the scanner software.

Language tasks were presented visually using goggles fitted to the head coil (NordicNeuroLab Visual System) which were connected to a computer running E-Prime 2 (Psychology Software Tools, Inc., Pittsburgh, USA). Stimuli were presented in block design. Ten scans were acquired per activation and baseline condition with an inter-scan interval of 3 s. For verb generation, 96 one- and two-syllable nouns were presented with an inter-stimulus-interval of 2.5 s. Patients were instructed to covertly produce one verb per noun. In sum, 210 scans were completed with an overall duration of 10.5 min per subject. The baseline condition consisted of the covert repetitive reading of the non-sense syllables “La-Li-Lo,” aiming to activate motor language cortices only. All subjects were intensively trained by a neuropsychologist before fMRI data acquisition, using a different word list.

#### Wada test

Wada test was administered on two consecutive days, starting with the anaesthetization of the hemisphere ipsilateral to the seizure focus. Evaluation of language comprehension and production included the naming of pictures and objects, the reading of written words and sentences, the detection of semantical and syntactical errors, and the pointing to colored geometrical forms taken from parts 2 and 3 of the Token-Test (Orgass et al., 1982). The anesthetized hemisphere was considered dominant for language if an initially demonstrated speech arrest was followed by dys- and paraphasias, eventually leading to full recovery.

#### Neuropsychological testing

All patients underwent standard neuropsychological evaluation before and 6 months after surgery. In order to assess naming functions, Boston Naming Test (BNT, Kaplan et al., 1983) was performed which consisted of 60 line drawings of non-animated objects presented in the order of increasing difficulty. According to Kaplan et al. (1983), test results are considered divergent if more than 12 errors occurred, corresponding to an error rate of >20%. A post-operative change in more or equal to five errors (8.33%) is considered significant (Sawrie et al., 1996).

### Data analysis

MEG and fMRI analysis were done blindly in patients with regard to the clinical parameters including Wada test outcome and amongst fMRI and MEG. As measurements in controls were aimed to define optimal parameters which were then fixed and used for the analysis of the patient group, MEG and fMRI analyses were not done blindly in control subjects.

#### MEG

MEG data analysis was done using MATLAB version R2021a (The Mathworks, Inc., Natick, MA) and the Field Trip package (Oostenveld et al., 2011) version 20210128.

##### Preprocessing

Data were cleaned from heartbeat and 50 Hz power line noise using cleaning methods from Tal and Abeles (2013). Data were visually inspected for jumps and bad channels, and respective time segments and channels were rejected. Data were epoched into trials with a post-stimulus interval of 1,500 ms and a pre-stimulus interval of 1,000 ms. Subsequently, data were demeaned using a baseline interval from 0 to 500 ms before stimulus onset, and bandpass filtered from 1 to 45 Hz. Residual external noise was removed using independent component analysis (ICA) of reference channels (Hanna et al., 2020). Successively, ICA was administered once again, and the topography and time course of components were visually inspected for eye blinks and other remaining artifacts. In case of a small number of eye blinks, single trials containing eye blinks were rejected from the original data. In other cases, the corresponding component was removed from the original data.

Data of patient no. 8 showed a low signal-to-noise ratio due to dental work within the lower frequency range affecting the evoked response. In this case, an additional high-pass-filter was applied at a frequency of 12 Hz before administering ICA and removing components with eye blinks and other artifacts.

After cleaning, an average of 167 trials (Min = 102, Max = 186) per patient was used for further analysis. In controls, an average of 171 trials were usable for analysis (Min = 140, Max = 183).

##### Exploratory analysis in sensor space

Exploratory sensor-level analyses were done in healthy subjects with the aim to define the optimal time interval and frequency range yielding left language lateralization. The defined time interval and frequency range was subsequently used for lateralizing hemispheric language dominance in the patient group.

The baseline corrected and averaged individual evoked response was z-transformed based on the pre-stimulus interval and split into two separate datasets with 115 left and 115 right sensors, respectively. We subsequently calculated the grand average in controls in order to investigate event-related components and their time course.

**Figure 1:**
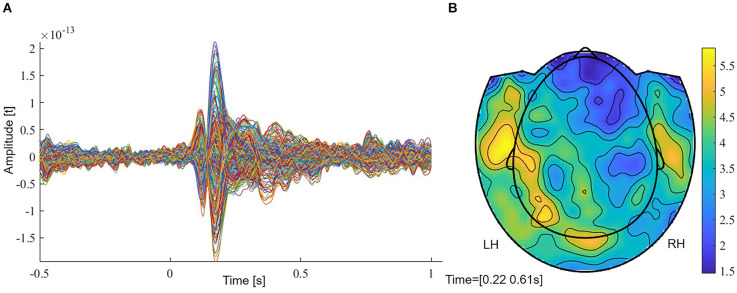
**(A)** Grand average of controls with two early (M100, M170) and a later component (M400). **(B)** Planar field distribution of the time-averaged activity of controls between 220 and 610 ms after stimulus onset. LH, left hemisphere; RH, right hemisphere.

The grand average of controls revealed two early components at around 70–140 ms (M100) and from 140 to 220 ms (M170), followed by a larger response between 220 and 610 ms (Figure 1A). In order to reveal significantly lateralized activity, we performed FDR-corrected non-parametric randomization testing of the root mean square of amplitudes for each time point between 220 and 610 ms. To test for a lateralized M400 component, we conducted a cluster permutation analysis implemented in FieldTrip for the visually determined time interval between 330 and 450 ms.

##### Frequency analysis

Explorative frequency analysis was done in controls in order to confirm the presence of event-related decreases in oscillatory power within the beta frequency range over left channel locations and to investigate its time course. We performed a wavelet analysis per subject for all available channel locations using Morlet wavelets for the time interval between −0.5 s and 1 s, a frequency range of 1–45 Hz in 1 Hz steps with a width of five cycles. Subsequently, the relative change in oscillatory power compared to the baseline for left and right sensors, respectively, was calculated. Its average over all the control subjects was used in order to select the time interval and the frequency range of interest.

##### Source estimation

Anatomical data were preprocessed using Fieldtrip. MEG data were co-registered to the T1 weighted images obtained prior to the fMRI session. The structural scans were aligned to the subject’s fiducial points and head shape obtained during MEG digitization. Brain tissues were extracted using SPM12 toolbox. A single shell model (Nolte, 2003) was created as volume conduction head model for each individual subject. For whole brain analysis, a template source model was used, consisting of a grid with a dimension of 18 × 21 × 18 cm and a resolution of 1 cm, encompassing 6,804 grid points. This resulted in 3,294 voxels inside the brain. The model was coregistered to individual MRI scans. For regions of interest analysis and statistics, the MNI-aligned template grid comprised a dimension of 17 × 21 × 18, a resolution of 1 cm, and a total of 6,426 grid points. A binary mask with 36 brain regions for a coarser region of interest analysis and with 18 regions for statistics [see section “Laterality Index (LI) and Regions of Interests (ROIs)” for details] was created from the Anatomical Automatic Labeling atlas (AAL, Tzourio-Mazoyer et al., 2002) and defined as grid points inside the brain, yielding in 651 and 318 voxels inside the brain, respectively. The source models were inversely warped to the subject-specific co-registered MRI.

Source estimation of power was done using Dynamic Imaging of Coherent Sources (DICS, Gross et al., 2001). This is a beamformer technique enabling the localization of oscillatory power in single-frequency bands. DICS has been successfully used for language lateralization and localization in a recent study in epilepsy patients using an auditory description naming and visual picture naming task (Youssofzadeh et al., 2020). Beamformer techniques also have been used successfully in language lateralization in patients using a verb generation task (Pang et al., 2011; Findlay et al., 2012; Wang et al., 2012). Beamformers allow source estimation of power for averaged and unaveraged data and were recommended as the best methods to compare MEG and fMRI results (Poline et al., 2010). Beamformers are particularly suited for analyzing patient data, as these methods attenuate spatially correlated noise (Cheyne et al., 2007).

**Figure 2:**
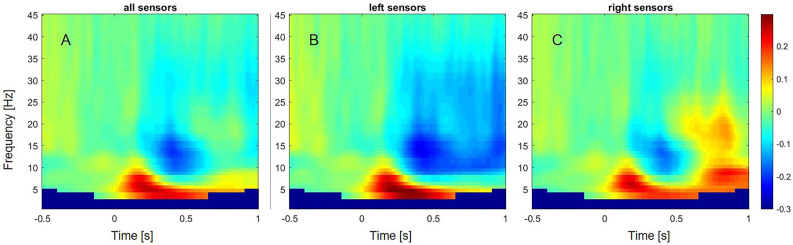
Event-related changes in oscillations across subjects and different frequency bands from 1 to 45 Hz. **(A)** Event-related oscillatory changes over all sensors. **(B)** Event-related oscillatory changes over left sensors. **(C)** Event-related oscillatory changes over right sensors.

In preparation of source estimation, trials were cut into baseline (0–700 ms pre-stimulus) and an experimental condition from 300 ms to 1 s, based on the observed beta desynchronization over left sensors in controls (see Figure 2). A time frequency analysis on the concatenated time series of the single trials was conducted using multiple tapers based on discrete prolate spheroidal sequences (DPSS, Slepian and Pollak, 1961) implemented in Fieldtrip. A central frequency of 24 Hz and a smoothing of 11 Hz were selected, based on the results of the exploratory sensor level analysis in controls. A common filter was computed including both conditions. The regulation parameter lambda was adjusted to 10% to account for the lower signal-to-noise ratio in the patient group. Subsequently, the percent change in event-related beta oscillations relative to baseline was calculated and interpolated to the individual MRI.

##### Statistical analysis

We examined in individual subjects if the change in beta source power after stimulus onset differed significantly from beta source power during the baseline interval. Statistical analysis was restricted to 18 brain regions involved in language processing which were defined based on the AAL atlas, encompassing a total of 318 voxels. We performed non-parametric Monte Carlo permutation tests (Maris and Oostenveld, 2007) of the source power of single trials for each voxel value with 10,000 permutations. A one-tailed dependent-sample *t*-test with a *p*-value of 0.05 was applied, resulting in a critical *t*-value of 1.65. Subsequently, we applied the cluster-correction method to account for the type I error rate. If there were not enough threshold surviving voxels left, the critical *t*-value was set to *t* = 1.97 equal to a *p*-value of 0.025 without correction for multiple comparisons.

#### fMRI

##### Preprocessing and statistical analyses

fMRI preprocessing and analysis were done with SPM12 (Wellcome Department of Imaging Neuroscience, London, UK). Raw DICOMs files were first converted to NifTI format. Volumes from each subject were realigned by using the first volume as the reference image. Preprocessing included motion using rigid body translation and rotation transformation and slice time correction, normalization of the co-registered functional and anatomical T1-weighted images into MNI space with respect to the MNI template, as well as spatial smoothing using a 4-mm Full Width Half Maximum Gaussian kernel.

Single subject first-level analyses were done contrasting task and control conditions with the application of a canonical hemodynamic response function without time or dispersion derivatives. The six head movement parameters that were generated during the realignment stage were included as motion covariates in the general linear model. A threshold of *p* < 0.001 (*t* = 3.13, uncorrected) with an extent to 10 voxels was applied to the individual *t*-maps. In controls, we additionally created individualized thresholds by taking the upper 10% of activated voxels for each subject, analogous to You et al. (2019) who reported that the top 10% of fMRI language activation significantly predicted post-operative naming decline. We compared laterality indices (LIs) between both approaches and interpreted similar LIs as an indication of a reliable threshold choice.

#### Laterality Index (LI) and Regions of Interests (ROIs)

fMRI and MEG t-maps were interpolated to fit the resolution of the AAL atlas. Laterality indices were then computed for three major regions involved in language processing: the frontal ROI encompassed pars opercularis and pars triangularis of the left and right inferior frontal gyrus (IFG; BA 44 and BA 45, “Broca’s area”) which is assumed to be engaged in executive speech and writing (Kamada et al., 2006), but was also found to be engaged in the processing of syntax and semantics (Goucha and Friederici, 2015). The temporo-parietal ROI consisted of the gyrus angularis, gyrus supramarginalis, and the superior temporal lobe (“Wernicke’s area,” WA), which is involved in the analysis and identification of linguistic sensory stimuli (Kamada et al., 2006). Recent studies show that a division between these two language areas is incorrect, as essential characteristics of language production and comprehension share the same underlying circuits, and lesions within Broca’s area impair not only language production but also language comprehension, whereas lesions in Wernicke’s area also affect language production (for review, see Hagoort and Indefrey, 2014). A decline in language functions such as naming ability may also be a consequence of temporal lobectomy (Davies et al., 1998; You et al., 2019), and lesions within the middle temporal gyrus may lead to semantic deficits and an impairment of word comprehension (for review, see Binder et al., 2009; Poeppel, 2014) we defined a third region of interest which included the middle and inferior temporal lobes and the temporal poles, referred to as “TIMP” in this study.

Laterality indices were calculated in two different ways: (1) based on the sum of threshold surviving *t*-values per left and right ROI, and (2) based on the count of threshold surviving voxels according to the formula: $LI=\frac{\sum tvaluesorvoxels_{left}-\sum tvaluesorvoxels_{right}}{\sum tvaluesorvoxels_{left}+\sum tvaluesorvoxels_{right}}$.

#### Comparison with Wada test

In order to enable comparison of MEG and fMRI laterality indices with trichotomous Wada test results, laterality coefficients are categorized into left, right, and bilateral activation by means of cut-off values. In the literature, a variety of cut-off values have been used, with ± 0.20 being the most frequently used value in fMRI (Bauer et al., 2014), and ± 0.10 in MEG (Papanicolaou et al., 2004; Tanaka et al., 2013; Raghavan et al., 2017; Kemp et al., 2018; Youssofzadeh et al., 2020). For the reason of comparability with other studies, a value of ±0.20 was chosen for fMRI and a value of ±0.10 for MEG. Left laterality was assumed at indices ≥0.2 in fMRI and at indices ≥0.1 in MEG, and right laterality at indices ≤ −0.2 in fMRI and at ≤ −0.1 in MEG. Values between 0.2 and −0.2 in fMRI and between 0.1 and −0.1 in MEG were considered bilateral.

Absolute and relative agreement rates were separately calculated for IFG, WA, and TIMP. As the sample size was small in our study, we did not calculate sensitivity and specificity rates. For statistics, the trichotomous language dominance categories “Left/Right/Bilateral” were reduced to the dichotomous categories “Left/Atypical” to increase the number of cases per cell. We used Cohen’s Kappa to assess the degree of agreement between the Wada test and fMRI and between the Wada test and MEG, respectively (Cohen, 1960). As Kappa can be inaccurate in case of imbalanced distribution of classes (Delgado and Tibau, 2019), we additionally calculated Matthews Correlation Coefficient as a performance metric (Boughorbel et al., 2017).

#### MEG-fMRI comparison

To examine the agreement of laterality indices between MEG and fMRI, unweighted Cohen’s Kappa was calculated for the trichotomous lateralization categories “left/right/bilateral” in all 24 patients. To examine the distance of maximum *t*-values between both methods in space, the coordinates of the maximum *t*-value in each ROI were located, and the Euclidean distance was calculated.

## Results

### MEG

#### Controls

##### Sensor level analysis

The grand average of controls revealed two early components at around 70–140 ms (M100) and from 140 to 220 ms (M170) (Figure 1A). A more widespread response was seen between 220 and 610 ms that showed stronger magnetic field amplitudes over left sensors (Figure 1B). Supplementary Figure 1 shows magnetic field distributions between 50 and 650 ms in steps of 50 ms.

**Figure 3:**
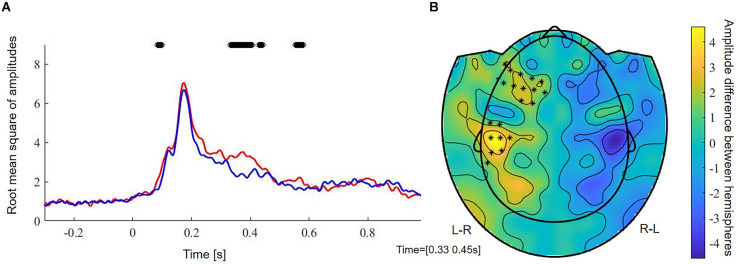
**(A)** Root mean square of amplitudes across subjects of left (red) and right (blue) sensors. Stars reflect significant time samples. **(B)** Raw effect and results of cluster-based permutation test between 350 and 450 ms. Stars reflect significant sensors. L-R, activation in left minus activation in right sensors; R-L, activation in right minus activation in left sensors.

FDR-corrected non-parametric randomization tests of the root mean square of the amplitude of the left and right sensors showed significant left lateralized intervals from 330 to 400 ms and from 550 to 576 ms (*p* < 0.05, corr., Figure 3A). Cluster-based permutation test within the latency range from 220 to 610 ms showed a significant difference between hemispheres which was most pronounced over frontal sensors (*p* = 0.012, corr.). Testing for an M400 effect in the latency range from 330 to 450 ms, the cluster-based permutation test revealed a significant difference between both hemispheres, which was most pronounced over frontal (*p* = 0.037, corr.) and temporal (*p* = 0.008, corr.) sensors (Figure 3B). No significant clusters were found for time intervals between 330 and 400 ms and between 550 and 576 ms.

##### Event-related oscillatory changes

In controls, a decrease of power within the alpha (8–13 Hz) and beta frequency range (13–35 Hz) over left sensors was observed, extending from around 300 ms to 1 s after stimulus onset (Figure 2). In contrast, an increase of power was observed within the beta frequency range between 700 and 900 ms over the right sensors. A power increase was also found for the theta (4–8 Hz) frequency band, ranging from 50 ms to 600 ms over left sensors, and extending to 1 s after stimulus onset over right sensors.

##### DICS beamformer results

**Figure 4:**
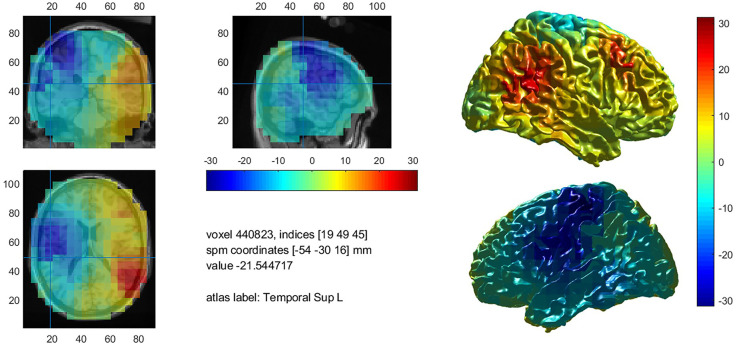
Mean decrease of beta band activity within the left hemisphere in controls.

When averaging the power maps of the control group, we observed an event-related beta desynchronization relative to the baseline within the left hemisphere and an event-related increase within the right hemisphere across subjects (Figure 4). Beta decrements were highest within the left post- (*M* = −22.9%, SD = 10.6) and precentral (*M* = −22.0%, SD = 10.2) region, followed by the Rolandic operculum (*M* = 20.4%, SD = 8.7). In language-related areas, beta desynchronization was highest within the pars opercularis of the inferior frontal gyrus (*M* = −20.1%, SD = 10.8), followed by the supramarginal (*M* = 17.3%, SD = 8.1), the superior temporal (*M* = −16.7%, SD = 9.5) and the middle temporal gyrus (*M* = −14.6%, SD = 9.7). A further peak of beta decrease (*M* = −18.7%, SD = 8.8) was seen for the Heschl gyrus which is part of the auditory cortex. A summary of mean beta decrements within left and right anatomical regions in controls is shown in Supplementary Figure 2.

##### Statistical analysis and laterality indices based on sum of threshold surviving *t*-values

There was no difference between laterality indices based on the sum of thresholded *t*-values and the count of thresholded voxels. Eight out of nine control subjects showed significant left lateralization in MEG with laterality indices of 1.0 for all ROIs when cluster-correction was applied to correct for multiple comparisons at a *p*-value of *p* < 0.05 (Table 2). One subject only showed significant left lateralization when leaving cluster-permutation tests uncorrected, still demonstrating laterality indices of 1.0 for all three regions of interest. Across subjects, an average of 42.6% of the left frontal ROI was significantly activated (SD = 20.0, range 5.0–57.7); left WA: 42.5% (range 13.0–62.6); left TIMP: 52.5% (range 0.95–66.3).

**Table 2:** MEG sum of thresholded and cluster corrected *t*-values (*p* < 0.05, cluster-corrected and “*” = uncorrected) in left and right ROIs and laterality indices in healthy controls.

		**Broca**			**Wernicke**			**TIMP**		
**Control no**	**EHI**	**left**	**right^a^**	**LI**	**left**	**right^a^**	**LI**	**left**	**right^a^**	**LI**
1	0.77	3,357.0	0.0	1.0	3,478.9	0.0	1.0	5,745.9	0.0	1.0
2	0.85	7,818.0	0.0	1.0	5,025.8	0.0	1.0	10,838.1	0.0	1.0
3	0.85	5,352.4	0.0	1.0	6,629.9	0.0	1.0	11,682.3	0.0	1.0
4	0.69	7,043.9	0.0	1.0	2,961.0	0.0	1.0	5,866.4	0.0	1.0
5	1.00	427.8*	0.0	1.0*	647.7*	0.0	1.0*	43.5*	0.0	1.0*
6	0.62	224.1	0.0	1.0	1,833.9	0.0	1.0	3,784.2	0.0	1.0
7	0.69	4,132.2	0.0	1.0	4,938.0	0.0	1.0	7,441.1	0.0	1.0
8	0.92	6,427.7	0.0	1.0	3,034.3	0.0	1.0	4,945.0	0.0	1.0
9	0.92	2,821.7	0.0	1.0	2,223.6	0.0	1.0	4,241.7	0.0	1.0
Mean	0.81	4,647.1	0.0	1.0	3,765.7	0.0	1.0	6,818.1	0.0	1.0
SD	0.13	2,515.4	0.0	0.0	1,627.5	0.0	0.0	2,966.8	0.0	0.0

^a^No voxels survived cluster corrected threshold in right ROI, i.e., the sum of thresholded *t*-values equals 0.0; EHI, Edingburgh Handedness Inventory; IFG, inferior frontal gyrus, WA, Wernicke Area; TIMP, temporal inferior and medial lobes plus temporal poles; LI, laterality index.

#### Patients

All eigth patients with left dominant language revealed by the Wada test were left lateralized in MEG in all ROIs (Table 3). Highest laterality was seen within the frontal language area (mean LI = 0.91, range 0.28–1.0), somewhat lower LIs were observed for WA with a mean LI of 0.77 (range 0.27–1.0), and a mean LI of 0.80 (range 0.39–1.0) for the TIMP. All six patients with atypical Wada test outcomes showed atypical laterality in MEG (Table 4). Patient 6 with partial left language comprehension showed bilateral laterality within the temporo-parietal ROIs. Patient 8 with bilateral language dominance but strong right hemispheric emphasis in the Wada test was completely right lateralized in MEG with high LIs of −1. LIs of all other right lateralized patients were congruent with Wada test.

**Table 3:** MEG sum of thresholded and cluster corrected *t*-values in left and right ROIs and laterality indices in patients with left language dominance revealed by Wada test.

			**IFG**			**WA**			**TIMP**		
**ID**	**Wada test**	**EHI**	**left**	**right**	**LI**	**left**	**right**	**LI**	**left**	**right**	**LI**
2	L	1.00	4,767.6	2,660.2	0.28	3,495.9	1,332.2	0.45	4,331.1	1,921.4	0.39
3	L	−0.70	2,266.8	0.0^a^	1.00	898.5	0.0^a^	1.00	2,574.1	0.0^a^	1.00
4	L	0.77	1,683.5	0.0^a^	1.00	1,059.6	0.0^a^	1.00	984.7	0.0^a^	1.00
12	L	0.25	4,122.3	0.0^a^	1.00	3,705.3	0.0^a^	1.00	5,830.0	0.0^a^	1.00
13	L	0.69	3,133.3	0.0^a^	1.00	2,232.2	736.0	0.50	4,487.4	1,580.3	0.48
17	L	1.00	10,022.9	0.0^a^	1.00	8,469.7	1,341.7	0.73	14,023.9	1,487.8	0.81
20	L	0.92	9,408.3	0.0^a^	1.00	5,911.1	1,149.1	0.67	8,661.9	1,482.7	0.71
23	L	1.00	4,466.0	0.0^a^	1.00	1,134.8	0.0^a^	1.00	5,075.7	0.0^a^	1.00
Mean		0.62	5,372.0	332.5	0.91	3,715.5	569.9	0.79	6,199.3	809.0	0.80
SD		0.59	3,143.4	940.5	0.25	2,689.0	636.8	0.24	4,129.2	875.4	0.25

^a^No voxels survived threshold in right ROI, i.e., the sum of thresholded *t*-values equals 0.0; ID, patient identification; EHI, Edingburgh Handedness Inventory; IFG, inferior frontal gyrus, WA, Wernicke Area; TIMP, temporal inferior and medial lobes plus temporal poles; LI, laterality index.

**Table 4:** MEG sum of thresholded and cluster corrected *t*-values in left and right ROIs and laterality indices in patients with atypical language dominance revealed by Wada test.

			**IFG**			**WA**			**TIMP**		
**Patient ID**	**Wada test**	**EHI**	**left**	**right**	**LI**	**left**	**right**	**LI**	**left**	**right**	**LI**
1	R	−0.08	3,433.5	5,345.1	−0.22	408.0	4,857.7	−0.85	951.2	8,036.6	−0.79
6	R/L*	0.92	520.6	2,789.0	−0.69	2,803.4	3,557.7	−0.12	4,064.1	3,879.9	0.02
8	Bil/R^†^	0.85	0.0^a^	5,242.5	−1.00	0.0^a^	2,834.5	−1.00	0.0^a^	3,550.3	−1.00
10	R	0.77	0.0^a^	1,567.4	−1.00	0.0^a^	35.5	−1.00	0.0^a^	1,267.2	−1.00
14	R	−0.69	0.0^a^	5,063.4	−1.00	0.0^a^	1,778.8	−1.00	0.0^a^	4,326.2	−1.00
21	R	0.92	3,956.3	6,696.0	−0.26	489.4	1,933.9	−0.60	3,313.5	6,539.9	−0.33
Mean		0.45	1,318.4	4,450.6	−0.69	616.8	2,499.6	−0.76	1,388.1	4,600.0	−0.68
SD		0.68	260.2	1,893.1	0.37	1,093.8	1,655.5	0.35	1,835.2	2,382.3	0.43

ID, patient identification; EHI, Edingburgh Handedness Inventory; IFG, inferior frontal gyrus, WA, Wernicke Area; TIMP, temporal inferior and medial lobes plus temporal poles; LI, laterality index; *right language dominance with partial left language comprehension; ^†^bilateral language dominance with strong right hemispheric emphasis; ^a^no voxels survived cluster corrected threshold in right ROI, i.e., the sum of thresholded *t*-values equals 0.0.

Cohen’s Kappa revealed 100% agreement (*k* = 1.0, *p*_s_ < 0.001) for IFG, WA, and TIMP. Matthew’s correlation coefficients were 1.0 for all three regions of interests.

A total of 24.4% percent of Broca’s area was significantly activated in patients who showed a clear left language dominance in MEG (*n* = 15). In 45.8% of all patients, the maximum *t*-value within the analysis was situated within Broca’s area. Supplementary Figure 3 shows an example activation map of patient 1.

### fMRI

#### Controls

There was no significant difference between LIs based on an activation threshold of *p* < 0.001 extended to a cluster of 10 voxels and LIs based on the individual upper 10% of activation. Further reports are limited to the former, non-individualized activation threshold.

##### Comparison between LIs based on voxel count and sum of *t*-values

**Figure 5:**
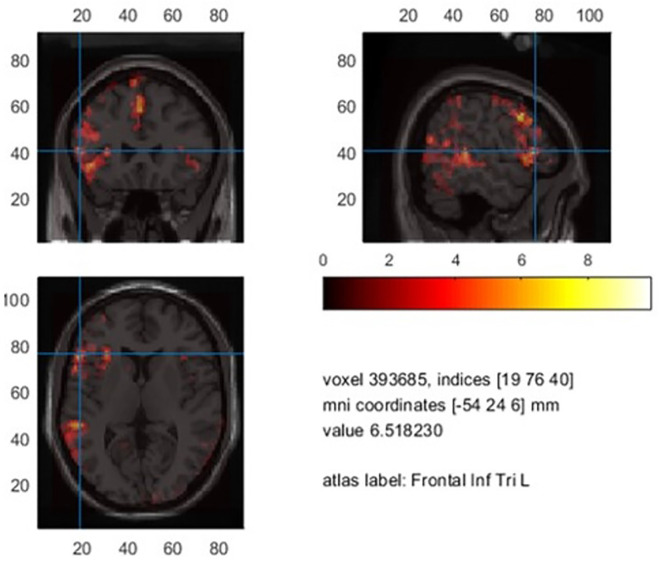
Mean significant *t*-values in controls.

On average, laterality indices encompassing all ROIs based on the sum of thresholded *t*-values were marginally higher (*M* = 0.64, range = −0.74–1.0) compared to the voxel count (*M* = 0.60, range −0.74–1.0) without yielding statistical significance. We limit the report of laterality indices based on the sum of *t*-values. The averaged BOLD signal in controls limited to the significant voxels is illustrated in Figure 5.

##### LIs based on threshold surviving *t*-values

Clear left laterality with LIs >0.4 was seen in all nine control subjects for IFG (range 0.55–1.0) as well as for TIMP (range 0.48–0.92). Only 4/9 subjects showed left lateralized results within WA (range −0.74–0.76). There were less significantly activated voxels within WA (*M* = 131.0, range 22.7–251.3) compared to IFG (*M* = 359.8, range 168.0–581.5) and TIMP (*M* = 260.2, range 41.5–492.8), with IFG being the smallest region of interest, encompassing 61.4% of the size of WA. On average, only 9.3% (range 3.9–15.5) of the left frontal ROI including pars triangularis and pars opercularis was significantly activated. Laterality indices in controls are found in Table 5.

**Table 5:** fMRI laterality indices based on the sum of thresholded *t*-values (*p* < 0.001, extended to 10 voxels) in healthy controls.

		**IFG**			**WA**			**TIMP**		
**Control no**	**EHI**	**left**	**right**	**LI**	**left**	**right**	**LI**	**left**	**right**	**LI**
1	0.77	2,278.3	125.4	0.90	606.6	133.1	0.64	1,804.5	137.7	0.86
2	0.85	1,345.0	388.9	0.55	442.2	391.1	0.06	941.7	63.7	0.87
3	0.85	2,819.3	101.6	0.93	492.0	92.0	0.68	1,610.2	122.1	0.86
4	0.69	1,148.1	0.0^a^	1.00	456.4	96.9	0.65	1,082.6	45.1	0.92
5	1.00	539.8	120.6	0.63	268.8	315.8	−0.08	135.6	43.4	0.52
6	0.62	1,135.0	37.5	0.94	286.7	109.3	0.45	851.4	57.2	0.87
7	0.69	1,205.9	7.1	0.99	181.5	162.4	0.06	728.5	257.9	0.48
8	0.92	2,136.6	82.2	0.93	1,116.0	149.9	0.76	2,526.5	190.4	0.86
9	0.92	1,799.2	64.1	0.93	11.6	78.9	−0.74	712.3	68.7	0.82
Mean	0.81	1,600.8	103.1	0.87	429.1	169.9	0.28	1,154.8	109.6	0.78
SD	0.13	710.5	116.5	0.16	314.2	109.2	0.50	713.3	74.6	0.17

Left/right, sum of threshold surviving *t*-values per left and right ROI. ^a^No voxels survived threshold in right ROI, i.e., the sum of thresholded *t*-values equals 0.0; EHI, Edinburgh Handedness Inventory; IFG, inferior frontal gyrus, WA, Wernicke Area; TIMP, temporal inferior and medial lobes plus temporal poles; LI, laterality index; left, sum of left *t*-values; right, sum of right *t*-values.

#### Patients

##### fMRI concordance with Wada test

The agreement between the Wada test and fMRI was highest for the IFG, with concordance in 12/14 patients (85.7%). Concordance between Wada test and fMRI WA and TIMP was achieved in 9/14 patients (64.3%). Seven out of eight (87.5%) patients with left language dominance in Wada test showed concordance for the IFG in fMRI (Table 6). Concordance with regards to the WA areas was low, with half of the patients showing agreement.

**Table 6:** fMRI laterality indices and the sum of threshold surviving *t*-values in patients with left language dominance revealed by Wada test (*p* < 0.001, extended to 10 voxels).

			**IFG**			**WA**			**TIMP**		
**ID**	**Wada test**	**EHI**	**left**	**right**	**LI**	**left**	**right**	**LI**	**left**	**right**	**LI**
2	L	1.00	1,887.6	247.0	0.77	205.5	1,163.7	−0.70	754.1	1,464.3	−0.32
3	L	−0.70	787.5	22.1	0.95	79.6	29.7	0.46	246.6	21.7	0.84
4	L	0.77	1,814.9	122.9	0.87	189.3	352.8	−0.30	643.8	610.5	0.03
12	L	0.25	1,131.4	72.9	0.88	474.1	160.6	0.49	754.4	234.8	0.53
13	L	0.69	1,316.8	152.3	0.79	83.5	103.7	−0.11	355.6	390.7	−0.05
17	L	1.00	1,851.4	44.1	0.95	51.2	139.0	−0.46	313.1	46.9	0.74
20	L	0.92	2,534.1	2,000.2	0.12	887.7	1,206.5	−0.15	1,487.4	1,283.9	0.07
23	L	1.00	3,663.2	415.0	0.80	942.9	108.9	0.79	873.3	111.6	0.77
Mean		0.62	1,873.4	384.6	0.77	364.2	408.1	0.00	678.5	520.6	0.33
SD		0.59	901.1	665.1	0.27	365.4	488.5	0.52	401.6	563.2	0.44

Left/right, sum of threshold surviving *t*-values per left and right ROI; ID, patient identification; EHI, Edinburgh Handedness Inventory; IFG, inferior frontal gyrus; WA, Wernicke Area; TIMP, temporal inferior and medial lobes plus temporal poles; LI, laterality index.

Five of six patients with atypical Wada test results showed concordance with fMRI for the IFG and TIMP (Table 7). When distinguishing between right and bilateral language outcome, discordance was seen in three patients. Patient 10 with right language dominance in the Wada test showed bilateral dominance in fMRI. Patient 6 with right language dominance and partial left language comprehension in Wada test showed bilateral language activation within the IFG, but clear right hemispheric laterality within the temporo-parietal regions. Patient 14 who was cognitively impaired only showed few significantly activated voxels within the left hemisphere. An adaptation of the critical value set to *t* = 1.65 (*p* < 0.05) did not change the direction of language dominance. When fMRI activation of this patient was rated by a trained radiologist using BrainVoyager Software (Goebel et al., 2006) used for clinical routine including an additional word fluency task, left lateralization was confirmed. All patients with atypical Wada test outcomes showed right lateralized activation within the WA. Cohen’s Kappa was 0.71 (*p* = 0.024, corr.) for IFG, indicating a substantial agreement, but not significant for WA (*k* = 0.46, *p* = 0.63, corr.) and TIMP (*k* = 0.34, *p* = 1.2, corr.). Matthew’s correlation coefficients were 0.71 for IFG, 0.45 for WA, and 0.55 for TIMP.

**Table 7:** fMRI laterality indices and sum of threshold surviving *t*-values in patients with atypical language dominance revealed by Wada test (*p* < 0.001, extended to 10 voxels).

			**IFG**			**WA**			**TIMP**		
**ID**	**Wada test**	**EHI**	**left**	**right**	**LI**	**left**	**right**	**LI**	**left**	**right**	**LI**
1	R	−0.08	2,552.3	3,843.3	−0.20	249.6	781.2	−0.52	537.5	815.9	−0.21
6	R/L*	0.92	1,213.4	926.0	0.13	19.3	714.4	−0.95	219.7	793.1	−0.57
8	Bil./R^†^	0.85	441.5	1,217.7	−0.47	127.6	738.9	−0.71	37.9	542.6	−0.87
10	R	0.77	1,328.6	1,585.6	−0.09	404.4	609.4	−0.20	807.2	548.7	0.19
14	R	−0.69	180.7	0.0^a^	1.00	0.0^a^	95.2	−1.00	19.6	118.2	−0.72
21	R	0.92	1,454.5	2,388.4	−0.24	136.4	689.8	−0.67	436.7	830.3	−0.31
Mean		0.45	1,195.2	1,660.2	0.02	156.2	604.8	−0.67	343.1	608.1	−0.41
SD		0.68	260.2	1,326.1	0.52	0.0	256.1	0.29	308.2	273.7	0.39

Left/right, sum of threshold surviving *t*-values per left and right ROI; ID, patient identification; EHI, Edinburgh Handedness Inventory; IFG, inferior frontal gyrus, WA, Wernicke Area; TIMP, temporal inferior and medial lobes plus temporal poles; LI, laterality index; *right language dominance with partial left language comprehension; ^†^bilateral language dominance with strong right hemispheric emphasis; ^a^no voxels survived threshold in right ROI, i.e., the sum of thresholded *t*-values equals 0.0.

Taken all 24 patients together, significantly fewer voxels were activated within the WA (*M* = 152.1, range 1.7–437.7) and TIMP (*M* = 246.9, range 1.5–867.5) compared to the IFG (*M* = 479.1, range 10.5–1,192.0; *F*_(2,71)_ = 12.73, *p* < 0.001). On average, 10.9% (range 1.4–20.2, SD = 0.8, *n* = 15) of the left IFG were significantly activated in left dominant patients revealed by fMRI. Supplementary Figure 3 shows an example activation map of patient 1.

With regard to lesion type, patients with focal cortical dysplasia and cortical malformation (*n* = 4) showed the least amount of threshold surviving voxels, with on average 91.6 voxels activated within the IFG, 18.7 voxels activated within WA and 29.6 voxels activated within TIMP. This corresponds to an average of 13% of the size of significant activation compared to patients with a different etiology. There was no further obvious association between the extent of lesion, site, or type of lesion and the extent of fMRI activation.

### Concordance between fMRI and MEG beta desynchronization

Cohen’s Kappa revealed 79.2% agreement of laterality indices in patients for IFG (*k* = 0.61, *p* = 0.003, corr.), an agreement of 56.6% for WA (*k* = 0.33, *p* = 0.36, corr.), and 65.2% agreement for TIMP (*k* = 0.40, *p* = 0.06, corr.).

There were less significantly activated voxels in fMRI compared to MEG (*t* = *p* < 0.001). In controls, on average, 9.34% (Min = 3.85%, Max = 15.49%) of Broca’s area was activated in fMRI, compared to 42.55% (Min = 4.77, Max = 57.72) in MEG.

**Figure 6:**
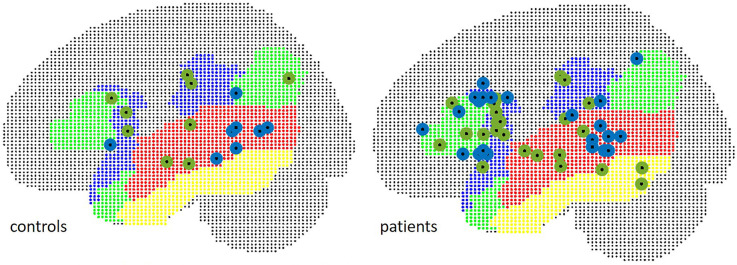
Localization of peak *t*-values in MEG (green) and fMRI (blue), separate for patients (right) and controls (left).

The localizations of the maximum *t*-value across all examined language relevant areas per subject were spread equally over inferior frontal and temporal ROIs (Figure 6): in 58.3% of patients, the maximum peak was situated within the IFG in fMRI, and in 45.8% of cases in MEG (Chi^2^ = 1.73, *p* = 0.19).

The mean Euclidean distance between the centroid of peak *t*-values within the frontal ROI in controls was 19.6 mm (range 11.8–28.7). In three patients, fMRI peaks were located within the contralateral hemisphere. The mean Euclidian distance for the remaining 21 patients was 21.5 mm (range 4.2–36.1).

**Figure 7:**
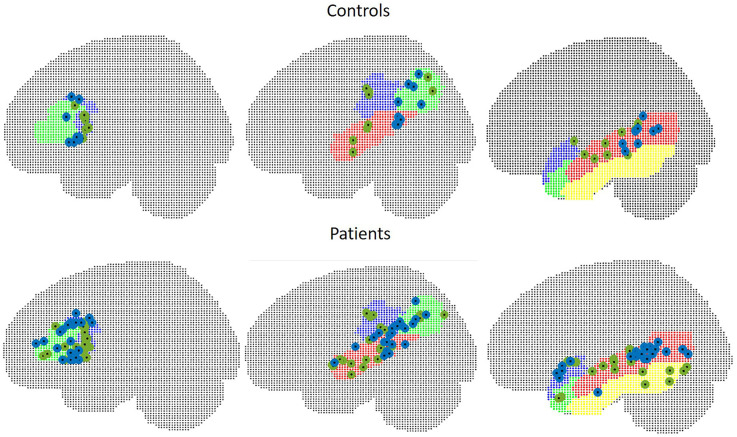
Peak intensity values of MEG (green) and fMRI (blue) in control subjects (upper figure) and in patients (lower figure). Left: frontal ROI, middle: temporo-parietal ROI, right: other temporal areas.

In WA, peak *t*-values were situated within the contralateral hemisphere in two control subjects and five patients. Euclidean distances were 34.5 mm in seven controls (range 19.6–50.5), and 33.8 mm in 18 patients (range 15.1–55.9) in TIMP, the mean Euclidean distance was 29.7 mm in controls (range 10.8–51.3) and 46.7 mm (range 9.1–103.6) in patients excluding five patients with peaks within the contralateral hemisphere. The locations of peak intensity values in patients and controls are illustrated in Figure 7.

### Postsurgical naming outcome

In two patients (ID 2 and ID 3), Boston naming test error scores were significantly increased at post-surgical evaluation six months after resection of parts of the language dominant anterior temporal lobe and amygdalohippocampectomy. Both patients already showed impaired naming functions pre-operatively. The decision regarding surgery was made due to an increase in seizure frequency in patient with ID no. 2. In patients with ID no. 3 and ID no. 4 (the latter with intact pre- and post-operative naming functions), the decision for surgery was grounded on memory dominance contralateral to the seizure focus revealed during the Wada test. Patient with ID no. 8 was operated on the non-dominant hemisphere and showed intact pre- and post-surgical naming functions.

## Discussion

There is no consensus on an optimal analysis method to determine language lateralization in presurgical evaluation using MEG (Youssofzadeh et al., 2020). The purpose of this study was to develop an efficient analysis method and to examine the concordance between MEG source localization of beta power desynchronization with fMRI and Wada test with regard to lateralization of expressive and receptive language areas using a visual verb generation task.

The present study adds to the current literature by providing further evidence for a robust lateralization of language based on beta power decrease using a verb generation task. In contrast to other studies, we pursued a two-step approach as the basis of our analysis strategy. We provided data of controls and patients, and built our analyses pipeline for MEG on parameters that were tested in controls and validated in patients. Only a few studies provide data from two modalities (fMRI, Wada test) to evaluate their lateralization results (e.g., Kamada et al., 2007; Foley et al., 2019).

In contrast to the state of the art in fMRI, only a few studies used statistical testing for thresholding language related source activation and determining language dominance in MEG (Hirata et al., 2010; Pang et al., 2011; Foley et al., 2019; Youssofzadeh and Babajani-Feremi, 2019; Youssofzadeh et al., 2020). A small number of studies applied Monte Carlo permutation testing for correction of multiple comparisons (Foley et al., 2019; Youssofzadeh et al., 2020), or applied cluster-correction to improve the sensitivity of statistics (Youssofzadeh et al., 2020).

We were able to achieve high laterality indices with MEG yielding agreement with Wada test in all 14 patients for inferior frontal and temporo-parietal language areas. fMRI agreed with Wada test in 12/14 of cases (85.7%) for expressive language, with non-significant agreement for receptive language areas. Concordance between laterality indices of MEG and fMRI was significant within the IFG, yielding 79.2% agreement and indicating a moderate to substantial agreement. Spatial agreement between MEG and fMRI varied considerably between subjects and brain regions and was highest within the IFG with a mean distance of the peak *t*-values of 21.5 mm (range 4.2–36.1) in patients.

### MEG

In concordance with the literature, we observed a beta decrease after stimulus onset within the language-related regions within the dominant hemisphere (Hirata et al., 2004, 2010; Fisher et al., 2008; Pang et al., 2011; Findlay et al., 2012; Foley et al., 2019; Youssofzadeh et al., 2020), and an increase within the non-dominant frontal and superior-parietal lobes (Sharma et al., 2021). The beta decrease was seen in controls between 300 and 800 ms and worked as the criterion to lateralize language in patients and controls. We can confirm findings from other studies reporting that beta decrease within 13–35 Hz is a robust marker for assessing language lateralization (e.g., Weiss and Mueller, 2012).

Besides oscillatory changes within the beta frequency range, we additionally observed an event related increase within the theta band and a decrement within the alpha band in both hemispheres. However, we did not systematically analyze these frequency patterns in this study. Changes within these frequency bands were also observed in other studies (Doesburg et al., 2016; Foley et al., 2019).

Localizing event-related beta desynchronization by means of DICS in combination with a cluster-corrected non-parametric permutation-test and a cut-off value of ±0.1, we achieved a complete agreement between Wada test and MEG for left and atypical language dominant patients for all three examined regions of interest involved in language processing. When distinguishing between right and bilateral language dominance, one patient (ID 8) with bilateral language dominance and strong right hemispheric emphasis showed right lateralized MEG with LIs of 1.0 in all ROIs. fMRI results were in line with MEG and also showed a clear right hemispheric laterality. This is unusual and studies more often report that bilateral or right lateralized Wada results tend to produce bilateral language representations in fMRI (Bauer et al., 2014). We cannot rule out that the Wada test anaesthetization within the right hemisphere declined preterm, and a right lateralization would represent the true language dominance in this patient.

In contrast to former studies that found language areas within the frontal lobe to be less activated, independent of the usage of receptive or expressive language tasks (for review, see Pirmoradi et al., 2010), we found 43% of the size of Broca’s area to be significantly activated in controls, and 24.4% in clearly left dominant patients revealed by MEG. In 45.8% of patients, the maximum *t*-value of all ROIs was situated within the IFG. As we also observed considerable activation within the Rolandic region in controls and patients, it cannot be ruled out that peak values within the IFG are partly due to spillover activation coming from the Rolandic region. The good agreement between the Wada test and LIs showed that IFG activation seen in this study serves well as a marker for language lateralization, but might not be suited well to localize language-related cortices within the IFG.

In a recent language lateralization study by Kemp et al. (2018) in a comparable patient group using a similar verb generation task but different analysis methods, the authors found concordance between MEG and Wada test in only 61.5% using a dipole method and in 46.2% applying a vectorized Type I beamformer, using a broadband filter from 1 to 80 Hz. In an unpublished work in our laboratory, applying LCMV and SAM beamformer, as well as dipole localization with broadband filtered data between 1 and 45 Hz, led to concordance rates with the Wada test in a similar range as reported by Kemp et al. (2018). While other studies were successful in applying these methods (for review, see Bowyer et al., 2020), we assume that a successful application might depend strongly on the selection of model parameters such as the time interval of covariance. A grounding of source localization on broadband filtered data might simultaneously capture excitatory and inhibitory oscillatory processes that might be prone to signal cancellation.

Kemp et al. (2018) presented their findings as a caution to viewing functional imaging as a replacement for the Wada test and that consensus on which imaging technique, which paradigms, and which analysis method to best apply clinically has to be established yet. From our experience, using beta desynchronization for language lateralization yielded the best results so far for the verb generation task in our laboratory. We support the position taken by Kemp et al. (2018) stating that functional imaging technology has to attain its full potential yet.

Of note, patients with low signal-to noise-ratio and dental work that tremendously affected the evoked response and challenged noise reduction during the preprocessing step were lateralized in line with the Wada test using DICS beamformer technique. Artifacts originating from magnetic dental fillings, implants, and braces are assumed to be caused by small vibrations and undetected motions of the jaw (Cheyne et al., 2007). The suppression of metal artifacts using beamformer techniques that are capable of dealing with correlated noise was shown in previous studies (Cheyne et al., 2007; Litvak et al., 2010).

### fMRI

In fMRI, left language lateralization in controls and concordance rates with the Wada test were highest within the IFG obtaining a substantial to strong agreement. Our results are in line with former studies that obtained the highest concordance rates with the Wada test for frontal language areas (Desmond et al., 1995; Binder et al., 1996; Bahn et al., 1997; Hertz-Pannier et al., 1997; Kamada et al., 2007; Youssofzadeh and Babajani-Feremi, 2019).

Regarding temporal language areas, the middle and inferior temporal cortices were lateralized to the left in controls, while the superior and parietal language areas tended to show bilateral or right lateralized activation. We found significant activation within the temporal lobes but contradicting laterality with regards to the Wada test in left dominant patients. The TIMP including the middle and inferior temporal lobes and the temporal poles yielded bilateral laterality while the WA including the STG tended to lateralize to the right. Similar results were reported by Youssofzadeh and Babajani-Feremi (2019) who found a right hemispheric activation of the STG. Other studies reported significantly fewer activated voxels within temporal language areas (Rutten et al., 2002), which is in line with our analysis. The use of sentence comprehension tasks for instance only activated a few voxels within the temporo-parietal region (Lehéricy et al., 2000; Rutten et al., 2002). In a study by of Billingsley-Marshall et al. (2007) comparing activation profiles between fMRI and MEG using a word recognition task, significantly fewer activated voxels within the superior temporal gyrus were obtained in fMRI. In another study by Kamada et al. (2007), activation spots within the superior temporal and the supramarginal gyrus were apparent in only 45% of investigated patients, using a categorization task. Elaborative language protocols seem to be needed to activate the receptive language areas (Rutten et al., 2002). An explanation for low detectability might be a shorter activation period of temporal compared to frontal language areas, with a decline as early as 500 ms after stimulus onset (Kunii et al., 2013). The temporal resolution of fMRI within 1–2 s due to slow coupling mechanisms between vascular processes and neuronal activity might account for lower temporal activation seen in BOLD contrast images (Rabrait et al., 2008).

Similar to other studies (for review, see Bauer et al., 2014), patients with atypical Wada test outcome more often demonstrated ambiguous results in fMRI: Two out of the six patients with atypical Wada result were misclassified in our study, and one patient with right language dominance in Wada test showed bilateral activation in fMRI. Patient no. 14 with right Wada test outcome showed left laterality within the IFG with few significantly activated voxels. The fMRI dataset was re-analyzed by a clinical radiologist using BrainVoyager QX software package (Brain Innovation B.V., Maastricht; The Netherlands) and lateralized visually yielding left laterality. However, this result must be taken with caution as the patient was cognitively impaired with difficulties following the instructions and stated to only have read the words out loudly. In MEG, she showed right lateralized language dominance. Furthermore, MRI revealed a fronto-parietal cortical dysplasia within the non-dominant hemisphere. Some studies suggest less reliable results in patients with extratemporal foci and larger lesions (Szaflarski et al., 2017).

Patient no. 6 who showed right language dominance and partial left language comprehension in the Wada test showed bilateral fMRI activation with left accentuation within the IFG. The clinical visual inspection of the fMRI resulted in bilateral activation with the right emphasis in line with the Wada test. MEG showed clear right language dominance in IFG. Differences between clinical reading and our computational LI approach might be caused by differences in pre-processing and statistical analysis. The clinical neuroradiologist analyzed fMRI using the BrainVoyager QX software package and estimated language dominance by looking at the activation of the entire brain using different thresholds and reducing or increasing the threshold more flexibly if laterality was unclear. In the present computational LI approach, LIs were based only on the activation within the ROIs. In addition, the way motion parameters were handled was not identical. The inclusion of motion covariates in the general linear model might have reduced the sensitivity of fMRI results in our study (Johnstone et al., 2006).

### Concordance and discrepancies between MEG and fMRI

The concordance between MEG and fMRI for the expressive language area found in this study along with the discordance regarding laterality and spatial agreement within the temporo-parietal language areas are congruent with former studies (Billingsley-Marshall et al., 2007; Kamada et al., 2007; Pang et al., 2011; Wang et al., 2012; Youssofzadeh and Babajani-Feremi, 2019). In line with other studies, a less robust lateralization for the temporal lobe was seen for fMRI. An identification of the receptive language areas would be of particular significance, as naming deficits pose the most frequent risks after resections within the dominant temporal lobe (Saykin et al., 1995; Schwarz et al., 2005) and a crossed language dominance with expressive and receptive language functions being located within contralateral hemispheres is not uncommon in epilepsy patients (Berl et al., 2014).

We support the view of Youssofzadeh and Babajani-Feremi (2019) who argued that discordances in lateralization may emphasize the need for multi-model integration of MEG and fMRI to obtain excellent predictive values. We advocate a more widespread complementary use of MEG for language lateralization in epilepsy centers. Bowyer et al. (2020) introduced an approach of first considering language lateralization with MEG, and consulting fMRI only in cases of inconclusive MEG results, while Wada testing being the last choice in cases of inconclusive MEG and fMRI results if no memory lateralization is required.

The extent of activation in patients and controls within the IFG was smaller in fMRI compared to MEG which is in contrast to other language studies displaying a robust activation of Broca’s area with fMRI (Desmond et al., 1995; Binder et al., 1996; Bahn et al., 1997; Hertz-Pannier et al., 1997; Kamada et al., 2007). It is also in contrast to reports that the identification of Broca’s area with MEG being more challenging (Pirmoradi et al., 2010; Pang et al., 2011). One language study directly compared MEG and fMRI using a verb generation task and found strong fMRI patterns in the IFG and less pronounced MEG activations (Pang et al., 2011). One possible explanation for the less robust fMRI activation in our study could be the use of different thresholds in MEG and fMRI. A cluster-permutation approach might be less stringent compared to using a thresholding *t*-value of 3.13 which was extended to a cluster of 10 voxels. However, less stringent thresholds in fMRI resulted in more bilateral laterality indices, resulting in less reliable lateralization. A more plausible explanation might be the differences in the baseline condition that may have contributed to a reduced number of threshold surviving voxels in fMRI. While we contrasted beta power against the pre-stimulus interval in MEG, the fMRI baseline condition consisted of the silent repetitive reading of nonesense syllables, aiming to activate motor language cortices only, but might have activated parts of the inferior frontal gyrus as well. This might have led to a reduced activation within Broca’s area due to an overlap of cortical activations between the control condition and the verb generation task. For instance, Goucha and Friederici (2015) found similar activation patterns for pseudowords compared to real words with the engagement of the inferior frontal gyrus. Papoutsi et al. (2009) report that Broca’s area as part of the sensory–motor integration system is directly involved in the generation or retrieval of the articulatory codes. Using an identical baseline in MEG and fMRI would have allowed a more direct comparison between the two modalities.

In contrast to MEG, participants were not able to control the presentation speed of the stimuli in fMRI. While the MEG stimulation protocol allowed for an adjustment in speed with regard to the abilities of the patient to process the task, the fixed inter-stimulus interval of 2.5 s in fMRI might have overstrained some patients leading to poor performance and hence less BOLD signal.

Differences between MEG and fMRI might also be attributed to the restriction of our MEG analysis to the beta frequency band. Studies showed that ERD of beta power and the BOLD signal are interrelated (Liljeström et al., 2015), but the BOLD signal also correlates with changes within the gamma and alpha bands (Zumer et al., 2010; Hall et al., 2014). Liljeström et al. (2015) argued to consider the entire MEG spectrum in order to analyze similarities between MEG and fMRI.

### Limitations

The small sample size of 24 patients of those only 14 patients obtained Wada testing limits the generalizability of our findings. We validated MEG and fMRI lateralization results with Wada results. Although the Wada test is seen as the gold standard, its relationship to the surgical outcomes has been limited (Loring and Meador, 2015). According to a review by Schmid et al. (2018), the Wada test does not provide satisfying predictive accuracy. There were too few patients that underwent surgery in our study to analyze the post-operative neuropsychological outcome which would be most appropriate for validation.

We only conducted one language task in our study that does not capture the full spectrum of language comprehension and production (Youssofzadeh and Babajani-Feremi, 2019). Temporal language areas were defined relatively coarse including broad regions, in particular within the temporal lobe. Our study comprises only a small part of the complex language system without taking language networks into account.

We used a covert task design which did not allow to control the performance of the task which would be of importance in particular in patients with low cognitive status. An overt design would overcome this limitation but led to a right-shift of laterality indices in a study by Berro et al. (2021). An additional control task as implemented in paradigms that are recommended by the American Society of Neuroradiology (Black et al., 2017) or the use of adaptive language mapping paradigms which showed strong lateralized activation maps in a study by Diachek et al. (2022) would allow to control the performance of the task.

We did not examine the consistency of MEG beta decrease in single subjects, while individual differences in the time course of beta decrements depending on age and cognitive ability might exist. As we localized the beta power decreases within a relatively broad time interval (300 ms to 1 s post-stimulus) that worked well for lateralization, we assume that we captured the stages of language processing in individual subjects.

We did not examine reproducibility and reliability in our study, as we initially intended to prove the suitability of our MEG analysis pipeline. A high test-retest reliability is mandatory in presurgical evaluation (Nettekoven et al., 2018; Agarwal et al., 2019) and will systematically be tested in a further study.

Due to the small sample size of patients with bilateral or right lateralized Wada test outcome, we summarized this patient group as “atypical” language lateralization. As there is a greater risk of language decline in unilateral dominant patients with ipsilateral surgery, an exact identification of bilateral or right-sided dominance would be of importance.

## Conclusion

Localizing the desynchronization of beta power with MEG using a verb generation task is a promising tool for language lateralization in pre-surgical evaluation of epilepsy patients. This study adds to the current literature by providing further evidence for robust lateralization of language with MEG using the DICS beamformer technique and cluster-based permutation testing for the identification of language dominance. A prospective study with a larger sample size including more atypically lateralized patients with information about the post-surgical outcome is needed to validate our findings.

## Data Availability Statement

The raw data supporting the conclusions of this article will be made available by the authors, without undue reservation.

## Ethics Statement

The studies involving human participants were reviewed and approved by the ethics committee of the Friedrich-Alexander-University Erlangen-Nuremberg. The patients/participants provided their written informed consent to participate in this study.

## Author Contributions

KH was responsible for the conceptualization, methodology, software, validation, formal analysis, investigation, data curation, project administration, visualization, and writing the original draft. YH was involved in software and formal analysis. JR was involved in investigation and formal analysis. NM participated in investigation. KW and MK were responsible for data curation. EP was involved in conceptualization, supervision, and funding acquisition. YH and AG provided support with MEG data analysis. AG, HH, MB, and AD provided the resources. JP was involved in funding acquisition. SR was responsible for conceptualization, software, validation, supervision, and funding acquisition. All authors contributed to the article and approved the submitted version.

## Funding

This study was supported by the following programs: (1) Bavarian Equal Opportunities Sponsorship—Realisierung von Frauen in Forschung und Lehre (FFL)—Promotion Equal Opportunities for Women in Research and Teaching provided by the University of Erlangen; (2) German Academic Exchange Service (DAAD); and (3) The ELAN Fund of the University Hospital of Erlangen, Germany (funding no. 07.11.26.1).

## Abbreviations

Bil.: Bilateral
IFG: Inferior Frontal Gyrus
L: Left
LI: Laterality Index
R: Right
ROI: Region of Interest
TIMP: Region of interest including the middle and inferior temporal lobes and the temporal poles
WA: Wernicke’s Area.

## References

[B1] Agarwal S. Sair H. I. Gujar S. Pillai J. J. (2019). Language mapping with fMRI: current standards and reproducibility. Top. Magn. Reson. Imaging 28, 225–233. 10.1097/RMR.0000000000000216 31385902

[B2] Bahn M. M. Lin W. Silbergeld D. L. Miller J. W. Kuppusamy K. Cook R. J.. (1997). Localization of language cortices by functional MR imaging compared with intracarotid amobarbital hemispheric sedation. Am. J. Roentgenol. 169, 575–579. 10.2214/ajr.169.2.9242780 9242780

[B3] Bauer P. R. Reitsma J. B. Houweling B. M. Ferrier C. H. Ramsey N. F. (2014). Can fMRI safely replace the Wada test for preoperative assessment of language lateralisation? A meta-analysis and systematic review. J. Neurol. Neurosurg. Psychiatry 85, 581–588. 10.1136/jnnp-2013-305659 23986313

[B4] Berl M. M. Zimmaro L. A. Khan O. I. Dustin I. Ritzl E. Duke E. S.. (2014). Characterization of atypical language activation patterns in focal epilepsy. Ann. Neurol. 75, 33–42. 10.1002/ana.24015 24038442 PMC4209919

[B5] Berro D. H. Lemée J.-M. Leiber L.-M. Emery E. Menei P. Minassian A. T. (2021). Overt speech critically changes lateralization index and did not allow determination of hemispheric dominance for language: an fMRI study. BMC Neurosci. 22:74. 10.1186/s12868-021-00671-y 34852787 PMC8638205

[B6] Billingsley-Marshall R. L. Clear T. Mencl W. E. Simos P. G. Swank P. R. Men D.. (2007). A comparison of functional MRI and magnetoencephalography for receptive language mapping. J. Neurosci. Methods 161, 306–313. 10.1016/j.jneumeth.2006.10.020 17157917

[B7] Binder J. R. Desai R. H. Graves W. W. Conant L. L. (2009). Where is the semantic system? A critical review and meta-analysis of 120 functional neuroimaging studies. Cereb. Cortex 19, 2767–2796. 10.1093/cercor/bhp055 19329570 PMC2774390

[B8] Binder J. R. Swanson S. J. Hammeke T. A. Morris G. L. Mueller W. M. Fischer M.. (1996). Determination of language dominance using functional MRI: a comparison with the Wada test. Neurology 46, 978–984. 10.1212/wnl.46.4.978 8780076

[B9] Black D. F. Vachha B. Mian A. Faro S. H. Maheshwari M. Sair H. I.. (2017). American society of functional neuroradiology-recommended fMRI paradigm algorithms for presurgical language assessment. Am. J. Neuroradiol. 38, E65–E73. 10.3174/ajnr.A5345 28860215 PMC7963630

[B10] Boughorbel S. Jarray F. El-Anbari M. (2017). Optimal classifier for imbalanced data using matthews correlation coefficient metric. PLoS One 12:e0177678. 10.1371/journal.pone.0177678 28574989 PMC5456046

[B11] Bowyer S. M. Moran J. E. Weiland B. J. Mason K. M. Greenwald M. L. Smith B. J.. (2005). Language laterality determined by MEG mapping with MR-FOCUSS. Epilepsy Behav. 6, 235–241. 10.1016/j.yebeh.2004.12.002 15710310

[B12] Bowyer S. M. Zillgitt A. Greenwald M. Lajiness-O’Neill R. (2020). Language mapping with magnetoencephalography: an update on the current state of clinical research and practice with considerations for clinical practice guidelines. J. Clin. Neurophysiol. 37, 554–563. 10.1097/WNP.0000000000000489 33165228

[B13] Cheyne D. Bostan A. C. Gaetz W. Pang E. W. (2007). Event-related beamforming: a robust method for presurgical functional mapping using MEG. Clin. Neurophysiol. 118, 1691–1704. 10.1016/j.clinph.2007.05.064 17587643

[B14] Cohen J. (1960). A coefficient of agreement for nominal scales. Educ. Psychol. Meas. 20, 37–46. 10.1177/001316446002000104

[B15] Davies K. G. Bell B. D. Bush A. J. Hermann B. P. Dohan F. C. Jaap A. S. (1998). Naming decline after left anterior temporal lobectomy correlates with pathological status of resected hippocampus. Epilepsia 39, 407–419. 10.1111/j.1528-1157.1998.tb01393.x 9578031

[B16] Delgado R. Tibau X.-A. (2019). Why Cohen’s Kappa should be avoided as performance measure in classification. PLoS One 14:e0222916. 10.1371/journal.pone.0222916 31557204 PMC6762152

[B17] Desmond J. E. Sum J. M. Wagner A. D. Demb J. B. Shear P. K. Glover G. H.. (1995). Functional MRI measurement of language lateralization in Wada-tested patients. Brain 118, 1411–1419. 10.1093/brain/118.6.1411 8595473

[B18] Diachek E. Morgan V. L. Wilson S. M. (2022). Adaptive language mapping paradigms for presurgical language mapping. Am. J. Neuroradiol. 10.3174/ajnr.A7629. [Online ahead of print]. 36137653 PMC9575518

[B501] Doesburg S. M. Tingling K. MacDonald M. J. Pang E. W. (2016). Development of network synchronization predicts language abilities. J. Cogn. Neurosci. 28, 55–68. 10.1162/jocn_a_00879 26401810 PMC4884083

[B19] Doss R. C. Zhang W. Risse G. L. Dickens D. L. (2009). Lateralizing language with magnetic source imaging: validation based on the Wada test. Epilepsia 50, 2242–2248. 10.1111/j.1528-1167.2009.02242.x 19674060

[B20] Findlay A. M. Ambrose J. B. Cahn-Weiner D. A. Houde J. F. Honma S. Hinkley L. B. N.. (2012). Dynamics of hemispheric dominance for language assessed by magnetoencephalographic imaging. Ann. Neurol. 71, 668–686. 10.1002/ana.23530 22522481 PMC3380661

[B21] Fisher A. E. Furlong P. L. Seri S. Adjamian P. Witton C. Baldeweg T.. (2008). Interhemispheric differences of spectral power in expressive language: a MEG study with clinical applications. Int. J. Psychophysiol. 68, 111–122. 10.1016/j.ijpsycho.2007.12.005 18316134

[B22] Foley E. Cross J. H. Thai N. J. Walsh A. R. Bill P. Furlong P.. (2019). MEG assessment of expressive language in children evaluated for epilepsy surgery. Brain Topogr. 32, 492–503. 10.1007/s10548-019-00703-1 30895423 PMC6476853

[B23] Goebel R. Esposito F. Formisano E. (2006). Analysis of FIAC data with BrainVoyager QX: from single-subject to cortically aligned group GLM analysis and self-organizing group ICA. Hum. Brain Mapp. 27, 392–401. 10.1002/hbm.20249 16596654 PMC6871277

[B24] Goucha T. Friederici A. D. (2015). The language skeleton after dissecting meaning: a functional segregation within Broca’s Area. Neuroimage 114, 294–302. 10.1016/j.neuroimage.2015.04.011 25871627

[B25] Gross J. Kujala J. Hamalainen M. Timmermann L. Schnitzler A. Salmelin R. (2001). Dynamic imaging of coherent sources: studying neural interactions in the human brain. Proc. Natl. Acad. Sci. U S A 98, 694–699. 10.1073/pnas.98.2.694 11209067 PMC14650

[B26] Hagoort P. Indefrey P. (2014). The neurobiology of language beyond single words. Annu. Rev. Neurosci. 37, 347–362. 10.1146/annurev-neuro-071013-013847 24905595

[B27] Hall E. L. Robson S. E. Morris P. G. Brookes M. J. (2014). The relationship between MEG and fMRI. Neuroimage 102, 80–91. 10.1016/j.neuroimage.2013.11.005 24239589

[B28] Hanna J. Kim C. Müller-Voggel N. (2020). External noise removed from magnetoencephalographic signal using independent component analyses of reference channels. J. Neurosci. Methods 335:108592. 10.1016/j.jneumeth.2020.108592 32017976

[B29] Hertz-Pannier L. Gaillard W. D. Mott S. H. Cuenod C. A. Bookheimer S. Y. Weinstein S.. (1997). Noninvasive assessment of language dominance in children and adolescents with functional MRI: a preliminary study. Neurology 48, 1003–1012. 10.1212/wnl.48.4.1003 9109891

[B30] Hirata M. Goto T. Barnes G. Umekawa Y. Yanagisawa T. Kato A.. (2010). Language dominance and mapping based on neuromagnetic oscillatory changes: comparison with invasive procedures. J. Neurosurg. 112, 528–538. 10.3171/2009.7.JNS09239 19681682

[B31] Hirata M. Kato A. Taniguchi M. Saitoh Y. Ninomiya H. Ihara A.. (2004). Determination of language dominance with synthetic aperture magnetometry: comparison with the Wada test. Neuroimage 23, 46–53. 10.1016/j.neuroimage.2004.05.009 15325351

[B32] Holland S. K. Plante E. Weber Byars A. Strawsburg R. H. Schmithorst V. J. Ball W. S. (2001). Normal fMRI brain activation patterns in children performing a verb generation task. Neuroimage 14, 837–843. 10.1006/nimg.2001.0875 11554802

[B33] Janecek J. K. Swanson S. J. Sabsevitz D. S. Hammeke T. A. Raghavan M. E Rozman M.. (2013). Language lateralization by fMRI and Wada testing in 229 patients with epilepsy: rates and predictors of discordance. Epilepsia 54, 314–322. 10.1111/epi.12068 23294162 PMC3649863

[B34] Johnstone T. Ores Walsh K. S. Greischar L. L. Alexander A. L. Fox A. S. Davidson R. J.. (2006). Motion correction and the use of motion covariates in multiple-subject fMRI analysis. Hum. Brain Mapp. 27, 779–788. 10.1002/hbm.20219 16456818 PMC6871380

[B35] Kamada K. Kober H. Saguer M. Möller M. Kaltenhäuser M. Vieth J. (1998). Responses to silent Kanji reading of the native Japanese and German in task subtraction magnetoencephalography. Brain Res. Cogn. Brain Res. 7, 89–98. 10.1016/s0926-6410(98)00016-0 9714756

[B36] Kamada K. Sawamura Y. Takeuchi F. Kuriki S. Kawai K. Morita A.. (2007). Expressive and receptive language areas determined by a non-invasive reliable method using functional magnetic resonance imaging and magnetoencephalography. Neurosurgery 60, 296–306. 10.1227/01.NEU.0000249262.03451.0E 17290180

[B37] Kamada K. Takeuchi F. Kuriki S. Todo T. Morita A. Sawamura Y. (2006). Dissociated expressive and receptive language functions on magnetoencephalography, functional magnetic resonance imaging and amobarbital studies. Case report and review of the literature. J. Neurosurg. 104, 598–607. 10.3171/jns.2006.104.4.598 16619665

[B38] Kaplan E. Harold G. Sandra W. (1983). Boston Naming Test. Philadelphia, PA: Lea & Febiger.

[B39] Kemp S. Prendergast G. Karapanagiotidis T. Baker G. Kelly T. P. Patankar T.. (2018). Concordance between the Wada test and neuroimaging lateralization: influence of imaging modality (fMRI and MEG) and patient experience. Epilepsy Behav. 78, 155–160. 10.1016/j.yebeh.2017.09.027 29245083

[B40] Kundu B. Rolston J. D. Grandhi R. (2019). Mapping language dominance through the lens of the Wada test. Neurosurg. Focus 47:E5. 10.3171/2019.6.FOCUS19346 31473678 PMC8117406

[B41] Kunii N. Kamada K. Ota T. Kawai K. Saito N. (2013). Characteristic profiles of high gamma activity and blood oxygenation level-dependent responses in various language areas. Neuroimage 65, 242–249. 10.1016/j.neuroimage.2012.09.059 23032488

[B502] Lehéricy S. Cohen L. Bazin B. Samson S. Giacomini E. Rougetet R.. (2000). Functional MR evaluation of temporal and frontal language dominance compared with the Wada test. Neurology 54, 1625–1633. 10.1212/wnl.54.8.1625 10762504

[B42] Liljeström M. Kujala J. Stevenson C. Salmelin R. (2015). Dynamic reconfiguration of the language network preceding onset of speech in picture naming. Human Brain Mapp. 36, 1202–1216. 10.1002/hbm.22697 25413681 PMC4365727

[B43] Litvak V. Eusebio A. Jha A. Oostenveld R. Barnes G. R. Penny W. D.. (2010). Optimized beamforming for simultaneous MEG and intracranial local field potential recordings in deep brain stimulation patients. Neuroimage 50, 1578–1588. 10.1016/j.neuroimage.2009.12.115 20056156 PMC3221048

[B44] Loring D. W. Meador K. J. (2015). “The Wada test: current perspectives and applications,” in Handbook on the Neuropsychology of Epilepsy, eds Barr W. B. Morrison C. (New York, NY: Springer New York), 123–137.

[B45] Maris E. Oostenveld R. (2007). Nonparametric statistical testing of EEG- and MEG-data. J. Neurosci. Methods 164, 177–190. 10.1016/j.jneumeth.2007.03.024 17517438

[B46] Massot-Tarrús A. Mousavi S. R. Mirsattari S. M. (2017). Comparing the intracarotid amobarbital test and functional MRI for the presurgical evaluation of language in epilepsy. Curr. Neurol. Neurosci. Rep. 17:54. 10.1007/s11910-017-0763-9 28623489

[B47] Nadkarni T. N. Andreoli M. J. Nair V. A. Yin P. Young B. M. Kundu B.. (2015). Usage of fMRI for pre-surgical planning in brain tumor and vascular lesion patients: task and statistical threshold effects on language lateralization. Neuroimage. Clin. 7, 415–423. 10.1016/j.nicl.2014.12.014 25685705 PMC4310930

[B48] Nettekoven C. Reck N. Goldbrunner R. Grefkes C. Weiß Lucas C. (2018). Short- and long-term reliability of language fMRI. Neuroimage 176, 215–225. 10.1016/j.neuroimage.2018.04.050 29704615

[B49] Nolte G. (2003). The magnetic lead field theorem in the quasi-static approximation and its use for magnetoencephalography forward calculation in realistic volume conductors. Phys. Med. Biol. 48, 3637–3652. 10.1088/0031-9155/48/22/002 14680264

[B50] Oldfield R. C. (1971). The assessment and analysis of handedness: the Edinburgh inventory. Neuropsychologia 9, 97–113. 10.1016/0028-3932(71)90067-4 5146491

[B51] Oostenveld R. Fries P. Maris E. Schoffelen J.-M. (2011). FieldTrip: open source software for advanced analysis of MEG, EEG and invasive electrophysiological data. Comput. Intell. Neurosci. 2011:156869. 10.1155/2011/156869 21253357 PMC3021840

[B52] Orgass B. de Renzi P. Vignolo A. (1982). Token-Test: (Manual). Göttingen: Hogrefe.

[B53] Pang E. W. Wang F. Malone M. Kadis D. S. Donner E. J. (2011). Localization of Broca’s area using verb generation tasks in the MEG: validation against fMRI. Neurosci. Lett. 490, 215–219. 10.1016/j.neulet.2010.12.055 21195135 PMC3076374

[B54] Papanicolaou A. C. Simos P. G. Breier J. I. Zouridakis G. Willmore L. J. Wheless J. W.. (1999). Magnetoencephalographic mapping of the language-specific cortex. J. Neurosurg. 90, 85–93. 10.3171/jns.1999.90.1.0085 10413160

[B55] Papanicolaou A. C. Simos P. G. Castillo E. M. Breier J. I. Sarkari S. Pataraia E.. (2004). Magnetocephalography: a noninvasive alternative to the Wada procedure. J. Neurosurg. 100, 867–876. 10.3171/jns.2004.100.5.0867 15137606

[B56] Papoutsi M. Zwart J. A. d. Jansma J. M. Pickering M. J. Bednar J. A. Horwitz B. (2009). From phonemes to articulatory codes: an fMRI study of the role of Broca’s area in speech production. Cereb. Cortex 19, 2156–2165. 10.1093/cercor/bhn239 19181696 PMC2722428

[B57] Pirmoradi M. Béland R. Nguyen D. K. Bacon B. A. Lassonde M. (2010). Language tasks used for the presurgical assessment of epileptic patients with MEG. Epileptic Disord. 12, 97–108. 10.1684/epd.2010.0314 20497912

[B58] Poeppel D. (2014). The neuroanatomic and neurophysiological infrastructure for speech and language. Curr. Opin. Neurobiol. 28, 142–149. 10.1016/j.conb.2014.07.005 25064048 PMC4177440

[B59] Poline J.-B. Line G. Lahaye P.-J. (2010). “Combining neuroimaging techniques: the future,” in MEG: An Introduction to Methods, eds Hansen P. C. Kringelbach M. L. Salmelin R. (New York: Oxford University Press), 273–299.

[B60] Rabrait C. Ciuciu P. Ribés A. Poupon C. Le Roux P. Dehaine-Lambertz G.. (2008). High temporal resolution functional MRI using parallel echo volumar imaging. J. Magn. Reson. Imaging 27, 744–753. 10.1002/jmri.21329 18383267

[B61] Raghavan M. Li Z. Carlson C. Anderson C. T. Stout J. Sabsevitz D. S.. (2017). MEG language lateralization in partial epilepsy using dSPM of auditory event-related fields. Epilepsy Behav. 73, 247–255. 10.1016/j.yebeh.2017.06.002 28662463

[B62] Rich B. A. Holroyd T. Carver F. W. Onelio L. M. Mendoza J. K. Cornwell B. R.. (2010). A preliminary study of the neural mechanisms of frustration in pediatric bipolar disorder using magnetoencephalography. Depress. Anxiety 27, 276–286. 10.1002/da.20649 20037920 PMC2841221

[B63] Rutten G. J. M. Ramsey N. F. van Rijen P. C. van Veelen C. W. M. (2002). Reproducibility of fMRI-determined language lateralization in individual subjects. Brain Lang. 80, 421–437. 10.1006/brln.2001.2600 11896650

[B64] Sawrie S. M. Chelune G. J. Naugle R. I. Lüders H. O. (1996). Empirical methods for assessing meaningful neuropsychological change following epilepsy surgery. J. Int. Neuropsychol. Soc. 2, 556–564. 10.1017/s1355617700001739 9375160

[B65] Saykin A. J. Stafiniak P. Robinson L. J. Flannery K. A. Gur R. C. O’Connor M. J.. (1995). Language before and after temporal lobectomy: specificity of acute changes and relation to early risk factors. Epilepsia 36, 1071–1077. 10.1111/j.1528-1157.1995.tb00464.x 7588450

[B66] Schmid E. Thomschewski A. Taylor A. Zimmermann G. Kirschner M. Kobulashvili T.. (2018). Diagnostic accuracy of functional magnetic resonance imaging, Wada test, magnetoencephalography and functional transcranial Doppler sonography for memory and language outcome after epilepsy surgery: a systematic review. Epilepsia 59, 2305–2317. 10.1111/epi.14588 30374948

[B67] Schwarz M. Pauli E. Stefan H. (2005). Model based prognosis of postoperative object naming in left temporal lobe epilepsy. Seizure 14, 562–568. 10.1016/j.seizure.2005.09.001 16236531

[B500] Sharma V. V. Vannest J. Greiner H. M. Fujiwara H. Tenney J. R. Williamson B. J.. (2021). Beta synchrony for expressive language lateralizes to right hemisphere in development. Sci. Rep. 11:3949. 10.1038/s41598-021-83373-z 33597643 PMC7889886

[B68] Slepian D. Pollak H. O. (1961). Prolate spheroidal wave functions, fourier analysis and uncertainty—I. Bell Syst. Tech. J. 40, 43–63. 10.1002/j.1538-7305.1961.tb03976.x

[B69] Springer J. A. Binder J. R. Hammeke T. A. Swanson S. J. Frost J. A. Bellgowan P. S.. (1999). Language dominance in neurologically normal and epilepsy subjects: a functional MRI study. Brain 122, 2033–2046. 10.1093/brain/122.11.2033 10545389

[B70] Szaflarski J. P. Gloss D. Binder J. R. Gaillard W. D. Golby A. J. Holland S. K.. (2017). Practice guideline summary: use of fMRI in the presurgical evaluation of patients with epilepsy: report of the guideline development, dissemination and implementation subcommittee of the american academy of neurology. Neurology 88, 395–402. 10.1212/WNL.0000000000003532 28077494 PMC5272968

[B71] Tal I. Abeles M. (2013). Cleaning MEG artifacts using external cues. J. Neurosci. Methods 217, 31–38. 10.1016/j.jneumeth.2013.04.002 23583420

[B72] Tanaka N. Liu H. Reinsberger C. Madsen J. R. Bourgeois B. F. Dworetzky B. A.. (2013). Language lateralization represented by spatiotemporal mapping of magnetoencephalography. Am. J. Neuroradiol. 34, 558–563. 10.3174/ajnr.A3233 22878013 PMC3732392

[B73] Tzourio-Mazoyer N. Landeau B. Papathanassiou D. Crivello F. Etard O. Delcroix N.. (2002). Automated anatomical labeling of activations in SPM using a macroscopic anatomical parcellation of the MNI MRI single-subject brain. Neuroimage 15, 273–289. 10.1006/nimg.2001.0978 11771995

[B74] Wang Y. Holland S. K. Vannest J. (2012). Concordance of MEG and fMRI patterns in adolescents during verb generation. Brain Res. 1447, 79–90. 10.1016/j.brainres.2012.02.001 22365747 PMC3461218

[B75] Weiss S. Mueller H. M. (2012). “Too many betas do not spoil the Broth”: the role of beta brain oscillations in language processing. Front. Psychol. 3:201. 10.3389/fpsyg.2012.00201 22737138 PMC3382410

[B76] You X. Zachery A. N. Fanto E. J. Norato G. Germeyan S. C. Emery E. J.. (2019). fMRI prediction of naming change after adult temporal lobe epilepsy surgery: activation matters. Epilepsia 60, 527–538. 10.1111/epi.14656 30740666 PMC6401285

[B77] Youssofzadeh V. Babajani-Feremi A. (2019). Mapping critical hubs of receptive and expressive language using MEG: a comparison against fMRI. Neuroimage 201:116029. 10.1016/j.neuroimage.2019.116029 31325641

[B78] Youssofzadeh V. Stout J. Ustine C. Gross W. L. Conant L. L. Humphries C. J.. (2020). Mapping language from MEG beta power modulations during auditory and visual naming. Neuroimage 220:117090. 10.1016/j.neuroimage.2020.117090 32593799

[B79] Zumer J. M. Brookes M. J. Stevenson C. M. Francis S. T. Morris P. G. (2010). Relating BOLD fMRI and neural oscillations through convolution and optimal linear weighting. Neuroimage 49, 1479–1489. 10.1016/j.neuroimage.2009.09.020 19778617

