# Supplementary material for: Localization of beta power decrease as measure for lateralization in pre-surgical language mapping with magnetoencephalography, compared with functional magnetic resonance imaging and validated by Wada test
Source: Front Hum Neurosci. 2022 Oct 26;16:996989. doi: 10.3389/fnhum.2022.996989 (PMC9644652; doi:10.3389/fnhum.2022.996989)
Supplement: Supplementary file 1 [file Data_Sheet_1.docx]

Supplementary Material

# Supplementary Figures:


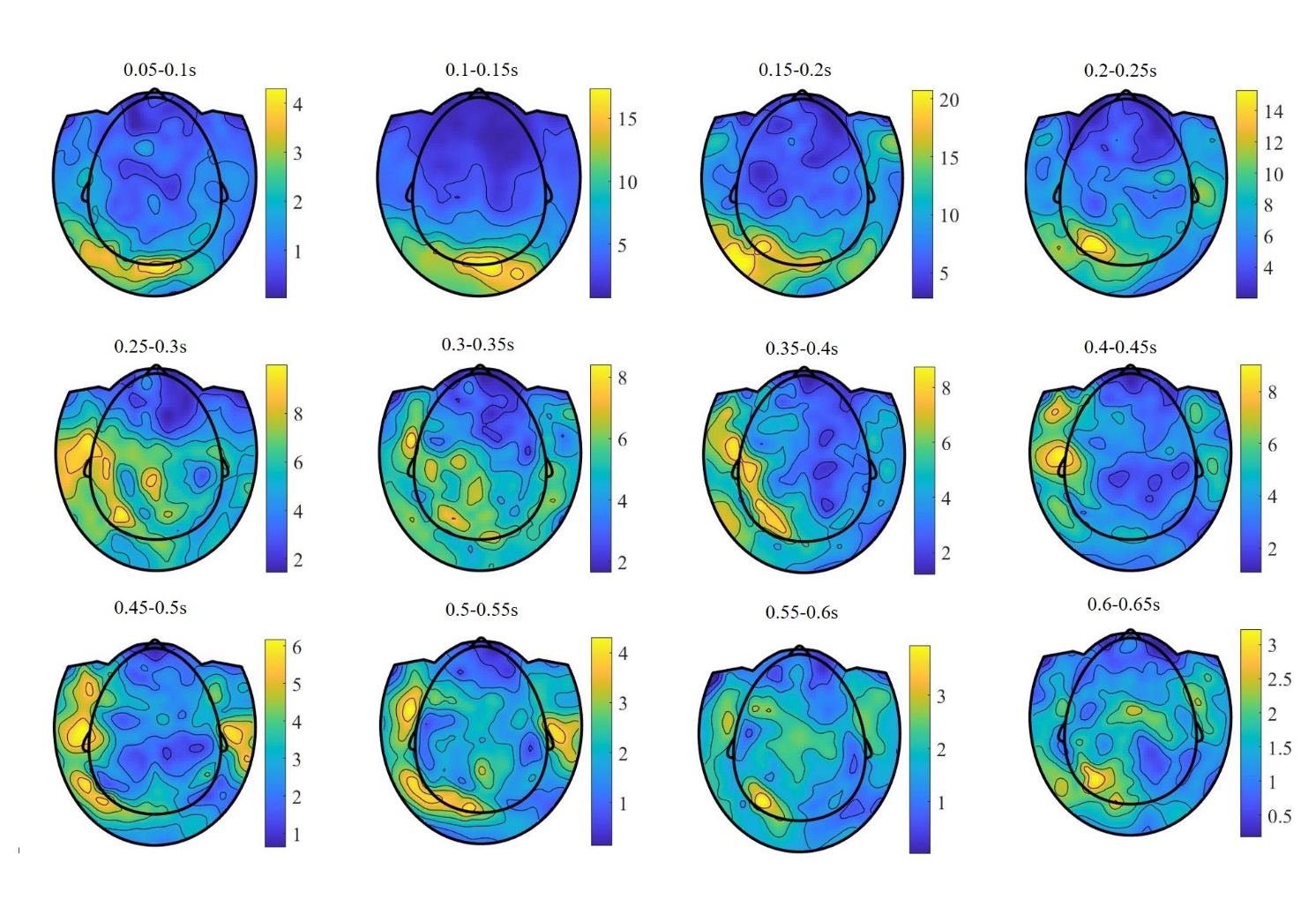


**Supplementary Figure 1.**. Mean planar field distributions in sensor space in controls between 50 and 650ms after stimulus onset in steps of 50ms.


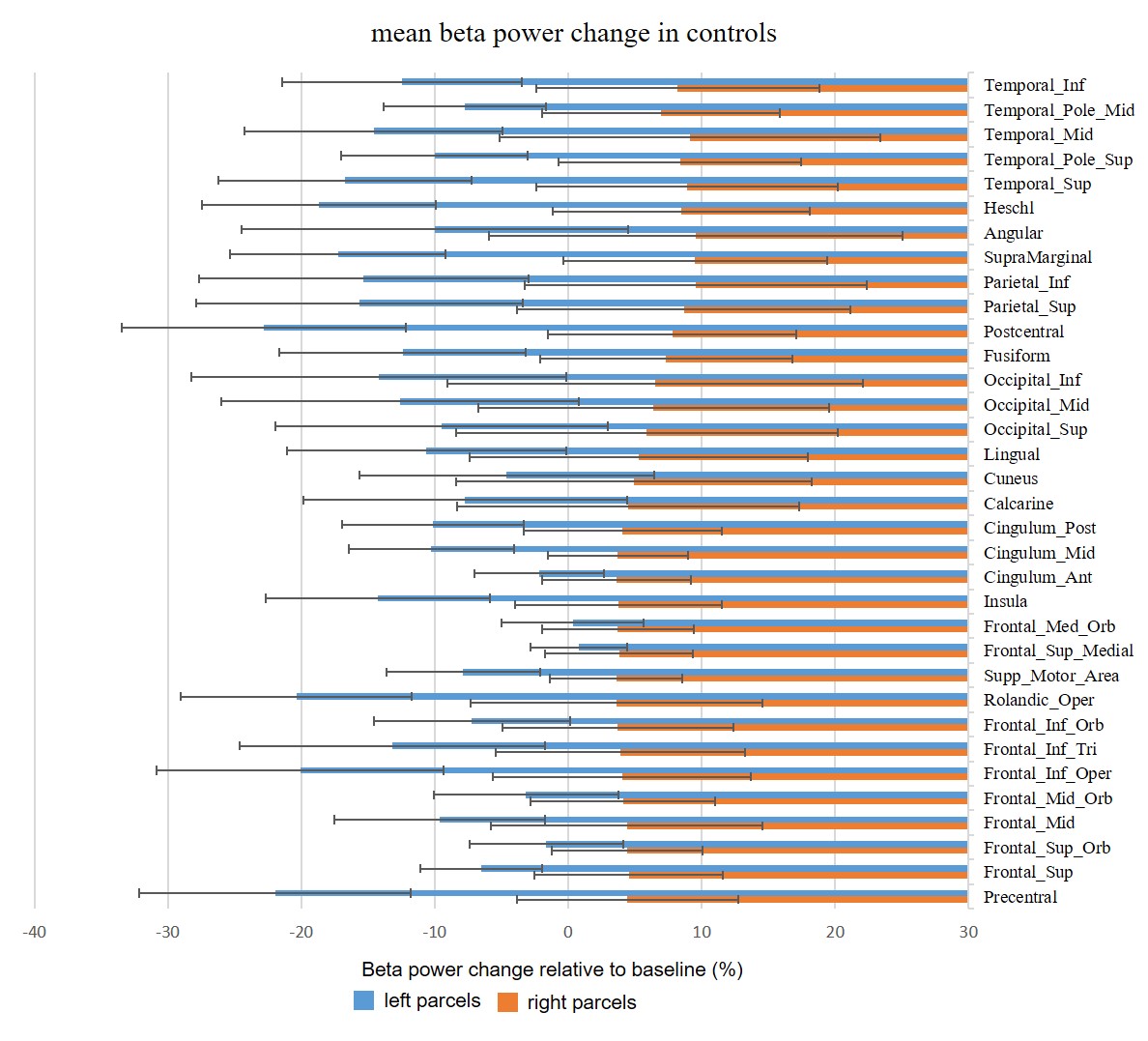


Supplementary Figure 2. Mean beta power change relative to baseline in controls in left and right anatomical parcels.


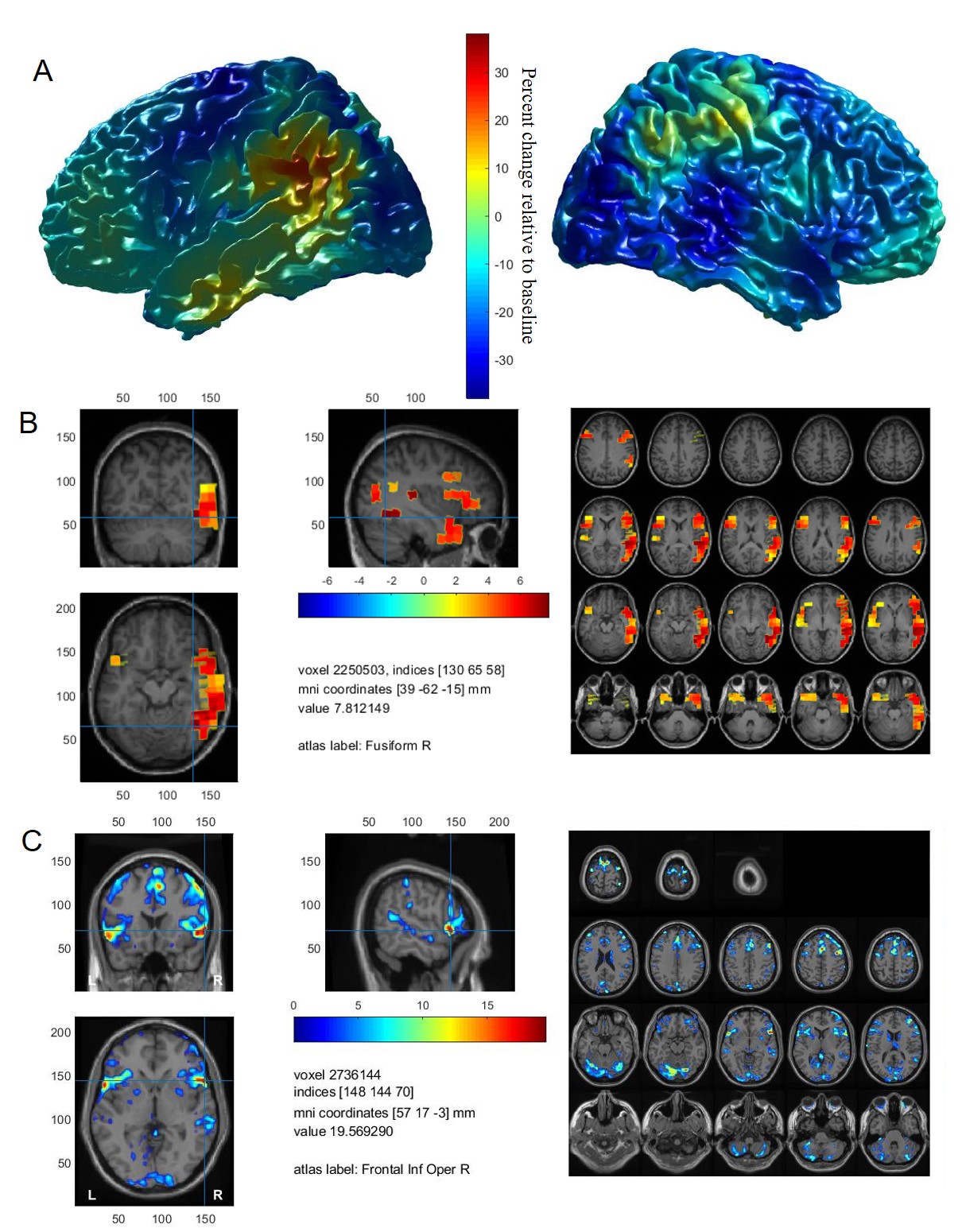


Supplementary Figure 3. Activation maps in patient 1 with right language dominance in Wada test. A. MEG localization of event related beta modulation between 0.3-1 sec after stimulus onset. Percent change relative to baseline (-0.7-0 sec). B. MEG result of cluster-based permutation testing which was restricted to the regions of interest. The peak t-value is situated within the right temporal lobe. C. fMRI statistical t-map thresholded at p<0.001, uncorrected with a cluster size of 10 voxels. Statistics were not restricted to ROIs. The peak t-value is situated within the pars opercularis of the right IFG.
